# Better Medications Adherence Lowers Cardiovascular Events, Stroke, and All-Cause Mortality Risk: A Dose-Response Meta-Analysis

**DOI:** 10.3390/jcdd8110146

**Published:** 2021-11-02

**Authors:** Mengying Liu, Guowei Zheng, Xiting Cao, Xinyu Chang, Ningning Zhang, Ge Liang, Anran Wang, Yan Yu, Yongli Yang, Yang Zhao, Xuezhong Shi, Dongsheng Hu, Jie Lu

**Affiliations:** 1 Department of Epidemiology and Health Statistics, College of Public Health, Zhengzhou University, Zhengzhou 450001, China; 18063131171@163.com (M.L.); 15128978244@163.com (G.Z.); lelush1122@163.com (X.C.); cxy1098805542@163.com (X.C.); ylyang377@126.com (Y.Y.); yzhao20@zzu.edu.cn (Y.Z.); xzshi@zzu.edu.cn (X.S.); dongsheng-hu@zzu.edu.cn (D.H.); 2 Department of Infection Prevention and Control, Huaihe Hospital of Henan University, Kaifeng 475000, China; zhangnn_2021@163.com; 3 Zhengzhou Central Hospital Affiliated to Zhengzhou University, Zhengzhou 450007, China; liangluyao315@163.com; 4 Department of Neurology, the First Affiliated Hospital of Zhengzhou University, Zhengzhou 450052, China; 17603719696@163.com; 5 Henan Center for Disease Control and Prevention, Society for Preventive Medicine, Zhengzhou 450018, China; hnzzyuy@163.com

**Keywords:** medication adherence, cardiovascular events, all-cause mortality, meta-analysis, dose-response

## Abstract

Aims: We investigated the association between vascular medication adherence, assessed by different methods, and the risk of cardio-cerebrovascular events and all-cause mortality. Methods: A meta-analysis with a systematic search of PubMed, Web of Science, EMBASE, and Cochrane databases from inception date to 21 June 2021 was used to identify relevant studies that had evaluated the association between cardiovascular medication adherence levels and cardiovascular events (CVEs), stroke, and all-cause mortality risks. Pooled relative risks (RRs) and 95% confidence intervals (CIs) were calculated using a random-effects meta-analysis. Restricted cubic splines were used to model the dose-response association. Results: We identified 46 articles in the dose-response meta-analysis. The dose-response analysis indicated that a 20% increment in cardiovascular medication, antihypertensive medication, and lipid-lowering medication adherence level were associated with 9% (RR: 0.91, 95% CI 0.88–0.94), 7% (RR 0.93, 95% CI: 0.84–1.03), and 10% (RR 0.90, 95% CI: 0.88–0.92) lowers risk of CVEs, respectively. The reduced risk of stroke respectively was 16% (RR: 0.84, 95% CI: 0.81–0.87), 17% (RR 0.83, 95% CI: 0.78–0.89), and 13% (RR 0.87, 95% CI: 0.84–0.91). The reduced risk of all-cause mortality respectively was 10% (RR: 0.90, 95% CI: 0.87–0.92), 12% (RR 0.88, 95% CI: 0.82–0.94), and 9% (RR 0.91, 95% CI: 0.89–0.94). Conclusions: A better medication adherence level was associated with a reduced risk of cardio-cerebrovascular events and all-cause mortality.

## 1. Introduction

Cardiovascular disease (CVD) is a disease of the heart and circulatory system. It is the leading cause of death and chronic disability worldwide, accounting for one-third of global deaths [1,2]. It is estimated that 23.6 million people will die from CVD by 2030 [3]. Stroke is one of the most important causes of mortality worldwide [4]. In 2014, the World Health Organization reported that out of the 17.5 million CVD deaths globally, about 6.7 million deaths were attributed to stroke [5]. An increased risk of cardio-cerebrovascular and subsequent adverse events is associated with a significant burden for the patients themselves, their family members, and healthcare systems, particularly in low- and middle-income countries [4,6]. Practical strategies for their prevention are imperative.

Cardiovascular medications remain the most common medical interventions worldwide for both the primary and secondary prevention of cardiovascular events (CVEs) through the modification of intermediate determinants of CVEs. Whereas a meta-analysis of almost 400,000 patients found that the adherence in patients taking these medications for both primary and secondary prevention of CVD was estimated at only 57% [7], a World Health Organization report emphasized that poor adherence to medication was a worldwide problem of striking magnitude [8]. Poor adherence reduces the effectiveness of essential medications and has been highlighted as a significant obstacle in achieving better patient outcomes [9,10,11]. Accumulating evidence supports that good medication adherence was associated with a lower risk of cardio-cerebrovascular events and all-cause mortality compared with poor adherence. However, the magnitude of the association has not been determined. Dongen [12] found that the antihypertensive medication (AHM) adherence levels were not associated with lower risks of recurrent vascular events among patients with stroke. In a meta-analysis of 21 studies, including participants across a range of conditions, good adherence was associated with a near halving of all-cause mortality compared with poor adherence [13]. Xu [14,15] showed a dose-response relationship between risk of stroke and AHM and statins adherence levels. An increment in AHM and statin adherence of 20% yielded a 9% and 8% lower risk of stroke, respectively.

Considering that whether the effect of adherence to cardiovascular medications for the prevention of cardio-cerebrovascular events and all-cause mortality were dose-dependent has not been determined. To date, no studies have systematically assessed the dose-response relationships of cardiovascular medications with the risk of cardio-cerebrovascular events and all-cause mortality. We targeted four cardiovascular medications, including lipid-lowering, antihypertensive, antidiabetics, and antithrombotic agents, and here report a systematic review and meta-analysis of the available studies to estimate the dose-response relationship between the risk of CVEs, stroke, and all-cause mortality and these four cardiovascular medication adherence. Such assessments are crucial for a better understanding of the relationship between cardiovascular medication adherence level and cardio-cerebrovascular events and all-cause mortality. Dose-response curves can provide more detailed medication adherence recommendations for reducing the risks of cardio-cerebrovascular events and all-cause mortality and provide valuable information for generating strategies to promote cardiovascular medication adherence to reduce CVD burden globally.

## 2. Methods

### 2.1. Literature Search

This review was conducted using a predefined protocol and following the Preferred Reporting Items for Systematic Reviews and Meta-Analyses (PRISMA) [16]. We searched electronic databases including PubMed, Web of Science, EMBASE, and Cochrane from inception date to 21 June 2021. We used combinations of medical subject headings and free text words that included search terms related to the exposure and medication groups, which were combined with search terms related to the outcomes (Supplementary Material S1). We identified articles eligible for further review by performing an initial screening of identified titles or abstracts, followed by a full-text review. The reference lists of all included articles and previous systematic reviews were manually searched. We contacted directly the authors of the retrieved papers for additional tabular data when required. In the case of multiple publications, the most recent and complete report was used.

### 2.2. Study Selection

Articles were considered for inclusion if the study (1) was published in English; (2) reported observational studies with a cohort or case-control design; and (3) reported risk estimates of lipid-lowering, antihypertensive, antidiabetics, and antithrombotic agents with CVEs (defined as any fatal or non-fatal coronary heart disease, myocardial infarction, heart failure, ischemic heart disease, or stroke or sudden cardiac death), stroke (defined as ischemic or hemorrhagic stroke, non-fatal or fatal stroke), or all-cause mortality (defined as mortality from any cause) outcomes.

### 2.3. Data Extraction and Quality Assessment

Two independent investigators (M.L and G.Z) collect relevant information included first author, publication year, location, population sources, design, characteristics of the population at entry, follow-up period, sex and age, methods used to assess adherence, overall level of adherence, reported levels of medication exposure, case number per category of medication exposure, total persons or person-years per medication category, odds ratios/relative risks (RRs)/hazard ratios for outcomes event with 95% confidence intervals (CIs) for each medication category, and covariates on which the analyses were adjusted. A study’s quality was evaluated using the Newcastle–Ottawa scale (NOS) [17], which has a maximum score of 9 points and summarizes eight aspects of each study. Any differences were resolved by discussion between the investigators.

### 2.4. Exposure Quantification

Medication adherence can be defined as the extent to which patients follow prescribed medication regimens at prescribed intervals and doses [18]. Epidemiological studies have used a wide range of tools to assess medication adherence, which can be broadly classified as indirect or direct. Adherence levels of indirect assessments are usually reported as the percentage of the prescribed doses of the medication taken by the patient over a specified period [11]. Moreover, they generally were evaluated by quantifying the implementation of the dosing program of patients, and the typical assessment methods mainly include the proportion of prescription drug-taking, the proportion of days with the correct dose, and the proportion taking the dosage on time, which was related to the time interval between prescribed continuous doses [19]. The proportion of days covered (PDC), medication possession ratio (MPR), cumulative medication adherence (CMA), and proportion of months covered (PMC) were similarly obtained by quantifying the proportion of days in which the correct dose was taken. We considered these measures to be of equal weighting. Medication adherence levels can vary along a continuum from 0.0% to 100.0%, and a higher percentage indicates a better adherence level.

### 2.5. Statistical Methods

Hazard ratios and odds ratios were assumed to approximate the same relative risk (RR). Summary RRs were calculated by pooling the study-specific estimates for various vascular medication types using a random-effects model which included between-study heterogeneity. If the number of cases in each exposure category was missing, we inferred these data from the total number of cases and the size of the reported effect. If each category did not report the exposed person-years or the number of participants, it was assumed to be of equal size [20]. When the reference category of medication adherence levels in the analysis was not the lowest, the method of Hamling [21] was adopted to transform the risk estimation. For each study, the median or average level of medication adherence for each category was assigned to the corresponding adjusted RR and 95% CI. If the median and average value of each category’s exposure level could not be obtained, the midpoint of each exposure category’s upper and lower boundaries was considered the average exposure level. Data where the results were reported by vascular medication types, and clinical outcomes were considered different studies. Data separately reporting the results by age were pooled with the fixed-effects model before inclusion in the meta-analysis. We used the method described by Greenland and Longnecker [22] for the dose-response meta-analysis and estimated study-specific trends and 95% CIs from the natural logs of the RRs and CIs across categories of medication adherence. Study-specific RR estimates were calculated per 20% increment of medication adherence and then pooled. We examined possible nonlinear associations by modeling medication adherence levels with a restricted cubic spline, with three knots at the 25th, 50th, and 75th percentile of the distribution [23]. Studies reporting risk estimates for at least three medication exposure levels for incident outcomes were included in the dose-response analysis. We calculated the *p*-value for nonlinearity by testing the null hypothesis of the second spline’s coefficient equal to zero.

Heterogeneity of RR estimates across studies were evaluated by the Cochran *Q* and *I*^2^ statistics [24]. For studies that included a dose-response meta-analysis, we performed subgroup analyses by gender, age, country/region, study quality, strategies for the assessment of medication adherence, follow-up time, levels of prevention, baseline population, and the covariates (adjusted for socioeconomic status, other co-medications, previous comorbidity, medication category, and number of medications). Sensitivity analysis was performed by excluding one study at a time to assess the stability of the results and potential sources of heterogeneity. The possibility of publication bias was evaluated using the Begg’s test [21] and visual inspection of funnel plots. If a statistically significant bias was found, the trim-filling method was used to adjust. *p* < 0.05 was considered statistically significant. All analyses involved the use of Stata 14 (Stata Corp., College Station, TX, USA).

## 3. Results

### 3.1. Literature Search and Characteristics of Studies

**Figure 1:**
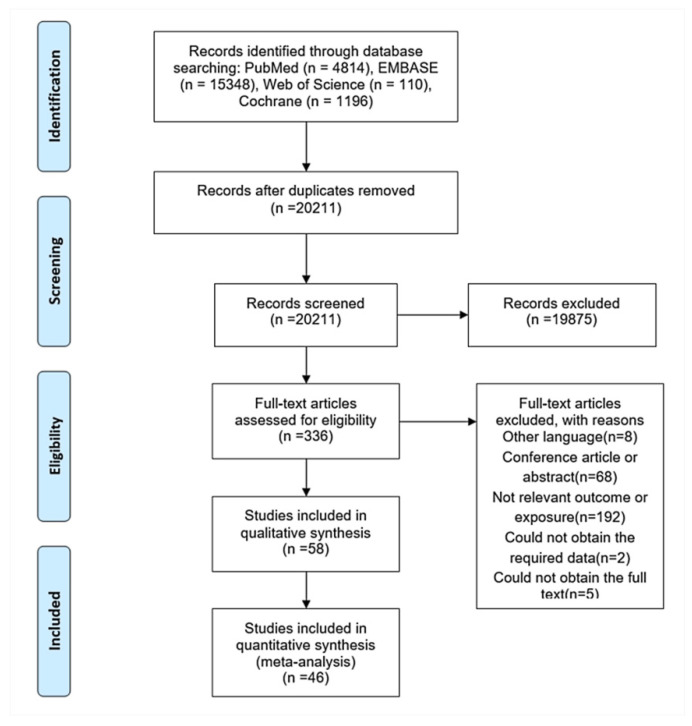
Flowchart of the literature search process.

A total of 20,211 citations were retrieved from the electronic search (Figure 1). After initial screening based on titles and abstracts, 336 articles were selected for further evaluation. In the full-text assessment, 46 of these articles met our inclusion criteria (Supplementary Material S2). The characteristics of the studies included are summarized in Table 1 and Table S1. Overall, data were available on 4051,338 unique participants with 187,306 CVEs, 51,794 stroke, and 175,050 all-cause mortality events collected over an average follow-up between 0.5 and 11 years. The average age of the participants was 60.1 years, and 56.0% of the participants were male. Thirty-one articles reported data on the adherence level, of which 54.0% participants had good adherence. Eighteen studies were based in Europe, 14 in North America, and 14 in Asia. Of these, 12 studies assessed adherence by the MPR; 31 by PDC; and 3 by other indirect measures including CMA, PDC and MPR, and PMC. The majority of the studies provided RRs for more than one medication. Among the studies identified, 35 reported on CVEs outcomes, 23 reported on stroke outcomes, and 26 reported on all-cause mortality. The data source and confounders adjusted in the multivariate analysis for each study were listed in Tables S2 and S3. The assessment of studies quality produced a mean NOS score of 7.9 (Table S4).

### 3.2. Medications Adherence and Any CVEs, Stroke, and All-Cause Mortality Analysis

**Figure 2:**
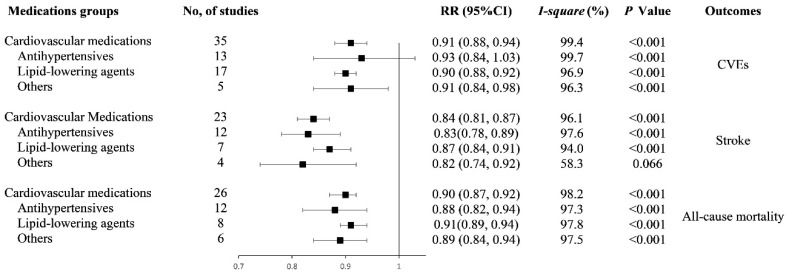
Forest plot of study-specific relative risk statistics for dose-response meta-analysis. Abbreviations: RR: relative risk; CI: confidence interval; CVEs: cardiovascular events; and other medications: multiple and antithrombotic medications.

We subsequently evaluated the dose-response relationship between any cardiovascular medication, each medications group adherence levels and risks of CVEs, stroke, and all-cause mortality. The results were summarized in Figure 2.

### 3.3. Cardiovascular Events

**Figure 3:**
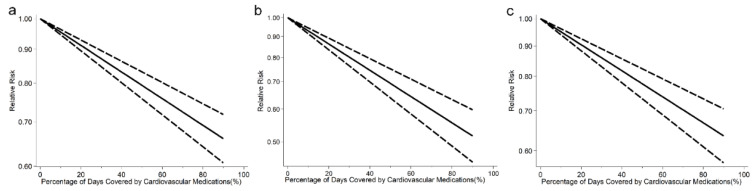
(**a**) Pooled dose-response analysis of cardiovascular medication adherence and cardiovascular events risk (solid line). (**b**) Pooled dose-response analysis of cardiovascular medication adherence and stroke risk (solid line). (**c**) Pooled dose-response analysis of cardiovascular medication adherence and all-cause mortality risk (solid line). Dashed lines represent the 95% CI.

Among the 35 studies reporting on CVEs outcome, we found a 20% increment in any cardiovascular medication adherence level was associated with a 9% lower risk of CVEs (RR: 0.91, 95% CI: 0.88–0.94, *I*^2^ = 99.4%, *p* < 0.001, Figure S1). The analysis of restricted cubic splines indicated a negative linear correlation between medication adherence and CVEs risk (*P_nonlinearity_* = 0.692, Figure 3a).

Thirteen studies and seventeen studies were included in the analyses of AHM and lipid-lowering medication adherence the risk of CVEs, respectively. The reduced risk of CVEs, respectively, were 7% (RR: 0.93, 95% CI: 0.84–1.03, *I*^2^ = 99.7%, *p* < 0.001) and 10% (RR: 0.90, 95% CI: 0.88–0.92, *I*^2^ = 96.9%, and *p* < 0.001) for 20% increment in AHM and lipid-lowering medication adherence levels (Figure S2). Results from the analysis of restricted cubic splines indicated a negative linear correlation between AHM, lipid-lowering medication adherence, and CVEs risk (*P_AHM nonlinearity_* = 0.556, *P_lipid-lowering_* *_medication nonlinearity_* = 0.757; Figure S3).

Four studies and one study analyzed multiple medication and antithrombotic medication adherence and CVEs risk, respectively. The combined RRs were 0.90 (95% CI: 0.83–0.99, *I*^2^ = 97.2%, *P_heterogeneity_* < 0.001) for multiple medication. For the all five studies, the combined RRs were 0.91 (95% CI: 0.84–0.98, *I*^2^ = 96.3%, *P_heterogeneity_* < 0.001) (Figure S4).

### 3.4. Stroke

In 23 studies with available data on stroke, we found a 20% increment in any cardiovascular medication adherence level was associated with a 16% lower risk of stroke (RR: 0.84, 95% CI: 0.81–0.87, *I*^2^ = 96.1%, *P_heterogeneity_* < 0.001, Figure S5). There was a negative association between medication adherence and stroke risk (*P_nonlinearity_* = 0.643, Figure 3b).

Twelve studies and seven studies were analyzed for an association of AHM, lipid-lowering medication adherence, and stroke risk, respectively. The reduced risks of stroke respectively were 17% (RR: 0.83, 95% CI: 0.78–0.89, *I*^2^ = 97.6%, *P_heterogeneity_* < 0.001) and 13% (RR: 0.87, 95% CI: 0.84–0.91, *I*^2^ = 94.0%, *P_heterogeneity_* < 0.001) for 20% increment in the AHM and lipid-lowering medication adherence level (Figure S6). There was a negative association between AHM, lipid-lowering medication adherence, and stroke risk (*P_AHM nonlinearity_* = 0.132, *P_lipid-lowering medication nonlinearity_* = 0.443; Figure S7).

Two studies analyzed the association of multiple medication adherence and the risk of stroke. Two studies were analyzed for the association of antithrombotic medication adherence and the risk of stroke. The combined RR was 0.82 (95% CI: 0.74–0.92, *I*^2^ = 58.3%, *P_heterogeneity_* = 0.066) (Figure S8).

### 3.5. All-Cause Mortality

Among the 26 studies reporting on all-cause mortality, we found a 20% increment in any cardiovascular medication adherence level was associated with an 10% lower risk of all-cause mortality (RR: 0.90, 95% CI: 0.87–0.92, *I*^2^ = 98.2%, *P_heterogeneity_* < 0.001, Figure S9). The analysis of restricted cubic splines indicated a negative linear correlation between medication adherence and all-cause mortality risk (*P_nonlinearity_* = 0.111, Figure 3c).

Twelve studies and eight studies reported the association of AHM, lipid-lowering medication adherence, and the risk of all-cause mortality. The reduced risk of all-cause mortality respectively were 12% (RR: 0.88, 95% CI: 0.82–0.94, *I*^2^ = 97.3%, *P_heterogeneity_* < 0.001) and 9% (RR: 0.91, 95% CI: 0.89–0.94, *I*^2^ = 97.8%, *P_heterogeneity_* < 0.001) for 20% increment in AHM and lipid-lowering medication adherence (Figure S10). There was a negative association between AHM adherence and all-cause mortality risk (*P_nonlinearity_* = 0.981, Figure S11a). A nonlinear negative association between lipid-lowering medication adherence and all-cause mortality risk (*P_nonlinearity_* < 0.001, Figure S11b).

Five studies and one study were analyzed for the association of multiple medication, antithrombotic medication adherence and the risk of all-cause mortality, respectively. The combined RR was 0.89 (95% CI: 0.83–0.95, *I*^2^ = 97.5%, *P_heterogeneity_* < 0.001) for multiple medication. For the all five studies, the combined RR was 0.89 (95% CI: 0.84–0.94, *I*^2^ = 96.9%, *P_heterogeneity_* < 0.001) (Figure S12).

### 3.6. Subgroup, Sensitivity Analyses, and Publication Bias 

We performed a subgroup analysis for the different outcomes by gender, age, country/region, study quality, strategies for the assessment of medication adherence, follow-up time, levels of prevention, baseline population, and the covariates (adjusted for socioeconomic status, other co-medications, previous comorbidity, medication category, and number of medications) (Figures S13–S15). There was no material difference in the pooled estimates associated with good adherence to cardiovascular medication among the different subgroup. On sensitivity analyses, removing one study at a time, the size or direction of the pooled estimates was similar for most results. Except for the studies reporting on AHM adherence and CVEs outcomes, when the studies [12,25,26,27] that were possible source of heterogeneity were eliminated from the pooled analysis, the heterogeneity was reduced, and the association changed to 0.89 (95% CI: 0.86–0.920, *I*^2^ = 76.0%, *P_heterogeneity_* = 0.003). There was no evidence of publication bias (*P*_Begg’s_ < 0.05), (Figure S16).

## 4. Discussion

Based on the available studies, we found an inverse association between higher medication adherence level and the risk of CVEs, stroke, and all-cause mortality. A 20% increment in cardiovascular medication, AHM, and lipid-lowering medication adherence level were associated with 9%, 7%, and 10% lower risk of CVEs, respectively. The reduced risk of stroke, respectively, was 16%, 17%, and 13%. The reduced risk of all-cause mortality respectively was 10%, 12%, and 9%.

The results were consistent with two recent meta-analyses [14,15], where they found an inverse association between AHM and statins adherence levels and the risk of stroke. It is worth noting that the current meta-analysis was the first study which used the method of dose-response meta-analysis to quantitatively investigate the relationship between cardiovascular medication adherence levels and the risk of CVEs, stroke, and all-cause mortality. Furthermore, the other obvious characteristics of this analysis were that it took multiple endpoints (including CVEs, stroke, and all-cause mortality) as outcome events, as well as taking multiple cardiovascular drugs (including lipid-lowering, antihypertensive, antidiabetics, and antithrombotic agents) into consideration. Meanwhile, compared with the previous studies, it included more comprehensive original studies and had a larger sample size. Thus, our finding might provide more comprehensive and robust evidence for clinical decision making and public health policy development to reduce the burden of death and cardiovascular disease.

The favorable consequences in those with good adherence levels to cardiovascular medication as observed in our review might have several explanations. First, cardiovascular medication not only could ameliorate hypertension and lower blood lipids but also have pleiotropic effects, such as antioxidant and anti-inflammation, improve vascular structure and function, endothelial stabilization, cerebral hemodynamics, antithrombotic action, and neuroprotective effects, which may be potential mechanisms underlying their preventative effects against CVEs and stroke [28,29,30,31,32,33,34]. Second, the adherence in these studies might simply be a surrogate marker of unmeasured confounders in these participants, with good adherence reflecting healthy behaviors or, conversely, comorbidities such as depression contributing to poor adherence in participants [13,35,36]. Our subgroup analyses comparing studies that adjusted socioeconomic variables and comorbidities with studies that did not yield any material difference in estimates. Despite the argued role of behavioral (or other unmeasured) attributes, the potential clinical benefits of good adherence to effective vascular medications are likely to be substantial and should not be underestimated.

With the restricted cubic spline model, we found a nonlinear inverse association between a higher lipid-lowering medication adherence level and the risk of all-cause mortality. Our study provided evidence that a better lipid-lowering medication adherence might be a clinically significant reduction in the risk for all-cause mortality: an important message for patients with lipid-lowering medication therapy. In addition, we found there was insufficient data to conduct a dose-response analysis for the relationship between antithrombotic or antidiabetic medication adherence level and the risk of CVEs, stroke, and all-cause mortality. Only two studies [37,38] reporting antithrombotic medication adherence were included in the dose-response analysis. In reviewing the literature, we noticed that few studies reported the association between cardiovascular medications adherence levels and relevant outcomes in vulnerable populations (e.g., cancer patients). Previous studies [39,40] reported on the significance of using cardiovascular medications to prevent cardiovascular disease in cancer patients. It is necessary to focus on the cardiovascular medications adherence status in the vulnerable populations in the future.

High heterogeneity was found in this study. Such high heterogeneity may be due to potential variations factors (such as drug dose intensities, different medication classes and quantities in each medication groups, and different baseline characteristics) in different studies. Considering the heterogeneity of the included studies, we performed sensitivity analyses and various subgroup analyses to discover the potential sources. Despite the heterogeneity only being partially explained, the potential benefits of good adherence to effective cardiovascular medications are substantial and should not be underestimated. Poor adherence to cardiovascular medications is highly prevalent across patient and cardiovascular medication classes, and the subsequent adverse health effects and healthcare burdens are enormous. A high cardiovascular drug adherence offers the possibility of a simple and cost-effective way to reduce a huge disease and healthcare burden. Multiple cardiovascular medications were considered to assess adherence levels and the risk of cardiovascular disease and all-cause mortality. Our findings highlight the overall importance of optimal adherence to cardiovascular medications in achieving better health outcomes. This will help shape clinical and public health strategies.

Poor adherence is still common, especially in low- and middle-income countries. Our study found that only 54% of participants in studies that reported levels of medication adherence reported good adherence (PDC ≥ 80%). The reasons for the situation of low medication adherence are multifaceted and closely related to society, economy, and patients themselves. A comprehensive approach should be taken to improve adherence to medication. For instance, key stakeholders in the health system including the government, research funders, health insurers, and the pharmaceutical industry should pay more awareness of this issue, by researching and promoting appropriate technologies for comprehensive medication adherence interventions. Individual online pharmacies with personalized management for different groups of people should be build and strengthening supervision and management. Meanwhile, relevant departments should increase education to raise patient awareness of medication adherence.

Adherence was a key factor associated with the effectiveness of all pharmacological therapies but was particularly critical for medications prescribed for chronic conditions. Improving medication adherence could be one of the most effective and efficient ways of improving health outcomes. Our study provides a complete evidence base to better understand the severe burden of nonadherence on cardiovascular disease and the economy and thus to develop strategies for improving cardiovascular drug adherence. Our meta-analysis has several limitations that should be considered when interpreting the results. Firstly, the current review combined only summary-level data of published studies; we were unable to assess trends in adherence levels over time. Secondly, a significant number of studies in this review were based on medication databases that used a wide range of definitions of adherence, where the level of adherence may vary substantially within the same population [41]. Currently, challenges remain in the approach to testing adherence, and it is difficult to agree on a gold standard for it [42]. The study included different adherence measurement indexes, which might be one of the main sources of heterogeneity. However, our results show that the subgroup estimates based on different adherence indexes were largely similar to the overall estimates, and to some extent, the robustness of the main results was confirmed. Thirdly, the medication adherence levels are not the only factor that influence the occurrence of cardiovascular events, strokes, and all-cause mortality. Other potential confounders, such as psychological factors or vulnerable populations, may reduce the strength of the association. Of the studies included in the meta-analysis, few studies took these confounders into consideration. Fourthly, the studies included in the meta-analyses involved different drug dose intensities, medication classes and quantities in each medication groups, and baseline characteristics (e.g., hypertensive patients and patients with CVD). It may affect the robustness of the association. When we confined the analysis to studies that adjusted for those factors, the inverse association between cardiovascular medication adherence levels and cardiovascular events, strokes, and all-cause mortality persisted.

## 5. Conclusions

Better medication adherence was associated with a reduced risk of CVEs, stroke, and all-cause mortality—particularly, the risk of stroke. Further prospective studies are needed to confirm the potential association between improved antithrombotic medication adherence levels and reduced CVEs, stroke, and all-cause mortality risk. As poor adherence remains a major barrier in achieving the full potentials of efficacious vascular medications, developing cost-effective measures to increase adherence should be considered a priority.

## Figures and Tables

**Table 1:** Summary characteristics of the unique studies included in this review.

Eligible Studies	Studies		Participants	
	*n*	%	*n*	%
Total unique Studies	46	100.0	4,051,338	100.0
Cohort	40	87.0	3,427,207	84.6
Nested Case-Control	6	13.0	624,131	15.4
Average Follow-Up (years), (range)	4.2	(0.5–11)	-	-
**Participants**				
Male (%), (range)	56	(1–100)	-	-
Average Age (years), (range)	60.1	≥18	-	-
**Location**				
Europe	18	39.1	1,137,158	28.1
North America	14	30.4	1,442,159	35.6
Asia	14	30.4	1,472,021	36.3
**Baseline Population**				
Healthy	1	2.2	84,262	2.1
Hypertensive	13	28.3	1,506,239	37.2
Hypercholesterolemia	10	21.7	1,379,902	34.1
Diabetic	2	4.3	95,070	2.3
Known Prior CVD	20	43.5	985,865	24.3
**Medication Group(s)**				
Lipid-Lowering Agents	21	45.7	2,271,122	56.1
Antihypertensive	17	37.0	1,643,330	40.6
Antiplatelet Agents	1	2.2	7,431	0.2
Multiple Vascular Agents	7	15.2	129,455	3.2
**Adherence Measure**				
MPR	12	26.1	1,728,753	42.7
PDC	31	67.4	2,226,750	55.0
Other	3	6.5	95,835	2.4
**Prevalence of Good Adherence**				
To Any CVD Medication	31	67.4	2,419,776	59.7
Percent	54.0	-	-	-
**Outcome Events**				
CVEs	35	76.1	187,306	4.6
Stroke	23	50.0	51,794	1.3
All-Cause Mortality Events	38	82.6	175,050	4.32

Abbreviations: MPR, medication possession ratio; PDC, proportion of days covered; Other: including proportion of months covered, PDC and MPR, and cumulative medication adherence. CVD: cardiovascular diseases; and CVEs: cardiovascular events.

## Data Availability

Not applicable.

## Supplementary Materials

The following are available online at https://www.mdpi.com/article/10.3390/jcdd8110146/s1, Figure S1: Forest plot of study-specific relative risk statistics for total cardiovascular diseases per 20% increment of cardiovascular medications adherence, Figure S2: Forest plot of study-specific relative risk statistics for total cardiovascular diseases per 20% increment of antihypertensive medication adherence (AHM) and lipid-lowering medications adherence, Figure S3: (a) Pooled dose-response analysis of antihypertensive medication adherence and total Cardiovascular Diseases risk (solid line). (b) Pooled dose-response analysis of lipid-lowering medication adherence and total Cardiovascular Diseases risk (solid line). Figure S4: Forest plot of study-specific relative risk statistics for total cardiovascular diseases per 20% increment of multiple medication and other medication (multiple and antithrombotic) adherence. Figure S5: Forest plot of study-specific relative risk statistics for stroke per 20% increment of medications adherence. Figure S6: Forest plot of study-specific relative risk statistics for stroke per 20% increment of antihypertensive medication adherence (AHM) and lipid-lowering medications adherence. Figure S7: (a) Pooled dose-response analysis of antihypertensive medication adherence and stroke risk (solid line). (b) Pooled dose-response analysis of lipid-lowering medication adherence and stroke risk (solid line). Dashed lines represent the 95% CI. Figure S8: Forest plot of study-specific relative risk statistics for stroke per 20% increment of other medication (multiple and antithrombotic) adherence. Figure S9: Forest plot of study-specific relative risk statistics for all-cause mortality per 20% increment of medications adherence. Figure S10: Forest plot of study-specific relative risk statistics for all-cause mortality per 20% increment of antihypertensive medication adherence (AHM) and lipid-lowering medications adherence. Figure S11: (a) Pooled dose-response analysis of antihypertensive medication adherence and all-cause mortality risk (solid line). (b) Pooled dose-response analysis of lipid-lowering medication adherence and all-cause mortality risk (solid line). Dashed lines represent the 95% CI. Figure S12: Forest plot of study-specific relative risk statistics for total cardiovascular diseases per 20% increment of multiple medication and other medication (multiple and antithrombotic) adherence. Figure S13: Subgroup analysis of dose response relation of risk of total cardiovascular diseases with cardiovascular medication adherence. Figure S14: Subgroup analysis of dose response relation of risk of stroke with cardiovascular medication adherence. Figure S15: Subgroup analysis of dose response relation of risk of all-cause mortality with cardiovascular medication adherence. Figure S16: (a) Publication bias test for the association between cardiovascular medications adherence and total cardiovascular diseases risk. (b) Publication bias test for the association between antihypertensive medications adherence and total cardiovascular diseases risk. (c) Publication bias test for the association between lipid-lowering medications adherence and total cardiovascular diseases risk. (d) Publication bias test for the association between cardiovascular medications adherence and stroke risk. (e) Publication bias test for the association between antihypertensive medications adherence and stroke risk. (f) Publication bias test for the association between lipid-lowering medications adherence and stroke risk. (g) Publication bias test for the association between cardiovascular medications adherence and all-cause mortality risk. (h) Publication bias test for the association between antihypertensive medications adherence and all-cause mortality risk. (i) Publication bias test for the association between lipid-lowering medications adherence and all-cause mortality risk. Begg’s test, *p* > |z| = 0.005 (continuity corrected), Table S1: Characteristics of the studies included in the meta-analysis, Table S2: The data source in each included study, Table S3: The confounders adjusted for the multivariate analysis in each included study, Table S4: Quality assessment of the included studies. Supplementary Material S1: Search strategy, Supplementary Material S2: Reference list for the studies included.
