# Supplementary material for: Better Medications Adherence Lowers Cardiovascular Events, Stroke, and All-Cause Mortality Risk: A Dose-Response Meta-Analysis
Source: J Cardiovasc Dev Dis. 2021 Nov 2;8(11):146. doi: 10.3390/jcdd8110146 (PMC8624664; doi:10.3390/jcdd8110146)
Supplement: Supplementary file 1 [file jcdd-08-00146-s001.zip › jcdd-1360667-supplementary.pdf]

Supplementary Material

Table S1 Characteristics of the studies included in the meta-analysis

Author	Study period	Country	Baseline population	Design	Medications	Prevalence of good adherence %	Duration of follow-up	Gender	Age（mean age）	Men %	Sample size	Case	Strategies for the assessment of adherence	outcomes
Korhonen 2015[1]	1995–2006	Finland	Diabetes patients	NCCS	Statins	49.3	3.9	M/W	64.2	62.9	8502	1703	PDC	Stroke
Bansilal 2016[2]	2010-2013	United States	MI and atherosclerotic disease patients	CS	Statins and ACE inhibitors	43	0.5	M/W	56.54	76.3	16991	836	PDC	CVE; Stroke
Karlsson 2017[3]	2007-2013	Sweden	Type 2 diabetes patients	CS	Lipid-lowering medications	NR	3.5	M/W	62	56.8	86568	14213	MPR	CVE; Stroke; All-cause mortality
Rodriguez 2019[4]	2013-2014	United States	ASCVD	CS	Statins	NR	2.9	M/W	21-85	98.4	347104	85930	MPR	All-cause mortality
Kim 2017[5]	2002-2012	Korea	AIS patients	CS	Statins	21.4	4.69	M/W	65-69	52.8	8001	2284	PDC	CVE
Corrao 2017[6]	2008-2012	Italy	Statins uesr	NCCS	Statins	NR	NR	M/W	>=60	51	273769	14633	PDC	CVE
Mathews 2018[7]	2007-2010	American	MI patients	CS	Multiple	NR	2	M/W	NR	48.5	19704	2757	PDC	CVE
Yeo 2020[8]	2010-2014	Singapore	IS patients	CS	Antithrombotic or statin	28.6	5	M/W	>=18	61	2299	178	PDC	Stroke; All-cause mortality

Rannanheimo 2015[9]	2001-2004	Finland	New statin user	CS	Statins	53	4	M/W	59.2	43.9	97575	12611	PDC	CVE; Stroke; All-cause mortality
Hickson 2019[10]	2008-2010	United States	MI patients	CS	Statins	50.4	0.95	M/W	>=65	45.7	101011	13274	PDC	All-cause mortality
Chen 2019[11]	2001-2005	China	AIS patients	CS	Antithrombotic Agents	26	2	M/W	67.8	56	7431	2238	PDC	CVE; Stroke; All-cause mortality
Ye 2013[12]	2005-2011	United States	Hyperlipidemia and/or type 2 diabetes mellitus patients	CS	Colesevelam HCl	18.7	1.6	M/W	58.1	44.9	42549	471	PDC	CVE
Molnar 2016[13]	2007–2011.	United States	CKD patients	CS	Multiple	NR	1.9	M/W	72	96	32348	18606	PDC and MPR	All-cause mortality
Chen 2016[14]	2002-2005	China	IS or TIA patients	CS	Statins	14.8	4.2	M/W	65	51.2	15408	5345	MPR	CVE
Phan 2019[15]	2006-2016	United States	MI patients	CS	Statins	81.4	4.3	M/W	85.4	2815	5629	3872	PDC	All-cause mortality
Shalev 2012[16]	1998-2009	Israel	New statin user	CS	Statins	28	5.79	M/W	57.82	42.32	171535	10159	PDC	CVE
Pittman 2011[17]	2008-2009	United States	All individuals with statin claim	CS	Statins	82.7	1.5	M/W	53	58.6	381422	25570	MPR	CVE
Shalev 2009[18]	1998-2006	Israel	new statins users with statin calim	CS	Statins	NR	5	M/W	57.6	49.2	229918	13165	PDC	All-cause mortality
Perreault 2009[19]	1999-2004	Canada	New statin users without prior CVD	NCCS	Statins	NR	1-6.5	M/W	63	38.6	115290	9294	MPR	CVE
Corrao 2010[20]	2002-2003	Italy	Healthy	CS	Statins	20.2	4.3	M/W	62	40.2	84262	1397	PDC	CVE

Rasmussen 2007[21]	1999-2003	Canada	AMI patients.	CS	Multiple	75	2.4	M/W	75.5	55.8	51310	10592	PDC	All-cause mortality
Wei 2004[22]	1994-1999	Scotland	CVD patients	CS	Multiple	31	3.7	M/W	66.3	54.2	865	395	MPR	CVE; All-cause mortality
Wei 2002[23]	1985-1995	Scotland	MI patients	CS	Statins	63.7	2.4	M/W	69.7	57.5	5590	2016	MPR	CVE; All-cause mortality
Esposti 2012[24]	2004-2006	Italy	New statins user	CS	Statins	41.1	0.5-3.5	M/W	66.5	51.1	19232	1579	PDC	CVE; Stroke; All-cause mortality
Perreault 2009[25]	1999-2004	Canada	New statins user	NCCS	Statins	55	2.95	M/W	63	41	112092	2593	MPR	All-cause mortality
Mazzaglia 2009[26]	2000-2005	Italy	Hypertensive patients	CS	AHM	8.1	4.6	M/W	62	41.6	18806	659	PDC	CVE
Herttua 2013[27]	1995-2007	Finland	Hypertensive patients	CS	AHM	NR	10	M/W	>=30	43.5	73527	26704	PDC	Stroke
Corrao 2011[28]	2000-2007	Italy	Antihypertensive drug user	CS	AHM	24.3	6	M/W	58.5	43.9	242594	12016	PDC	CVE
Krousel-Wood 2015[29]	2006-2011	United States	Hypertensive patients	CS	AHM	71.8	3.8	M/W	75	40.2	2075	240	MPR	CVE
Wong 2013[30]	2001-2012	China	New antihypertensive drug user	CS	AHM	55	5	M/W	ALL	45.1	218047	3825	PDC	All-cause mortality
Kim 2016[31]	2002-2010	South Korea	New Hypertensive patients	CS	AHM	31.3	8	M/W	>=20	46.6	33728	5225	CMA	CVE; Stroke; All-cause mortality
Herttua 2016[32]	1995-2007	Finland	Hypercholesterolemia patients	CS	AHM	NR	5.5	M/W	>=30	46	58266	532	PDC	Stroke

Esposti 2011[33]	2004-2006	Italy	New antihypertensive drug user	CS	AHM	41.5	1.9	M/W	60.2	48	31306	1263	PDC	CVE; Stroke; All-cause mortality
Yang 2016[34]	2007-2012	United States	Hypertensive patients	CS	AHM	46	6	M/W	50.3	32.7	59037	10041	MPR	CVE; Stroke
Liu 2009[35]	1999-2004	China	New Hypertensive patients	CS	AHM	NR	3.2	M/W	55.3	51.8	29759	1078	PMC	Stroke
Corrao 2015[36]	2005-2012	Italy	HF or hypertensive HF patients	NCCS	AHM	NR	6.6	M/W	67	54	76017	622	PDC	CVE
Yang 2017[37]	2008-2014	United States	Hypertensive patients	CS	AHM	60.8	5.8	M/W	69.9	36.3	155597	47198	PDC	CVE; Stroke
Kim 2016[38]	2007-2011	Korea	New Hypertensive patients	CS	AHM	57.7	3	M/W	58.8	47.5	564782	4655	MPR	CVE
Corrao 2017[39]	2007-2012	Italy	New antihypertensive drug user	NCCS	AHM	43.8	4	M/W	88	39.9	38461	14343	PDC	CVE; Stroke; All-cause mortality
Dongen 2019[40]	1994-2007	Finland	IS	CS	AHM	40	8.3	M/W	44	62.6	936	470	PDC	CVE; Stroke; All-cause mortality
Lee 2017[41]	2009-2013	Korea	Hypertensive patients	CS	AHM	68.8	NR	M/W	56.7	54.2	38520	957	MPR	Stroke
Kim 2018[42]	2002-2013	South Korea	Hypertensive patients with AHS	CS	AHM	49.5	4.45	M/W	62	47.8	1872	634	PDC	Stroke
Lavie 2021[43]	2009-2014	Israel	New statins user	CS	Statin	5.7	5	M/W	57.2	42.6	42767	4964	PDC	CVE; All-cause mortality
Lee 2020[44]	2003-2013	South Korea	dyslipidemia	CS	Statin	41.87	11	M/W	NR	43.03	107954	1143	PDC	All-cause mortality
Greenland 2020[45]	2003-2008	Australian	MI patients	CS	Statin; Beta-blocker	NR	1	M/W	77.2	57.5	5938	1575	PDC	CVE; All-cause mortality

Ahrens 2021[46]	2010-2015	German	MI patients	CS	Lipid-Lowering	66.8	2.9	M/W	66.7	68.7	14944	668	PDC	CVE

AHM: antihypertensive medications; MPR, medication possession ratio (no. of days of medication supplied within refill interval/no. of days in refill interval); PDC, proportion of days covered (no. of days with medication on-hand/no. of days in specified time interval); PMC: proportion of months covered by prescribed; CMA: cumulative medication adherence; CVD: cardiovascular disease; CS: cohort study; NCCS: nested case-control study; CAD: coronary artery disease; CHD: coronary heart disease; AMI: acute myocardial infarction; HF: heart failure; IS: Ischemic stroke; TIA: transient ischemic attack; AIS: acute ischemic stroke; AHS: acute hemorrhagic stroke; MI: myocardial infarction; ASCVD: atherosclerotic cardiovascular disease; CKD: chronic kidney disease; ACS: acute coronary syndrome; CVE: cardiovascular events; NR, not reported

Table S2 The data source in each included study

**Author**	**Data source**
Korhonen 2015	Finnish health registers
Bansilal 2016	Medical and pharmaceutical claims obtained from Aetna Commercial and Medicare Advantage population databases
Karlsson 2017	Swedish Prescribed Drug Register Swedish Prescribed Drug Register, National Diabetes Register, National Patient Register, Cause of Death Register, Longitudinal Integration Database for Health Insurance and Labour Market Studies
Rodriguez 2019	Veterans Affairs Health System
Kim 2017	National Health Insurance Service-National Sample Cohort.
Corrao 2017	Healthcare utilization databases of Lombardy,
Mathews 2018	Acute Coronary Treatment and Intervention Outcomes Network Registry-Get With The Guidelines
Yeo 2020	National Healthcare Group and Singapore Stroke Registry,
Rannanheimo 2015	Prescription, special reimbursement, and hospital discharge registers and registers of Statistics Finland.
Hickson 2019	US Medicare 2007 2011 data and prescription Part D claims from the CMS Chronic Conditions Data Warehouse
Chen 2019	Taiwan's National Health Insurance claims dataset
Ye 2013	MarketScan commercial and Medicare databases
Molnar 2016	US Renal Data System
Chen 2016	Taiwan Bureau of National Health Insurance database
Phan 2019	An integrated healthcare system in Southern California.
Shalev 2012	Maccabi Healthcare Services
Pittman 2011	Medco National Integrated database
Shalev 2009	Maccabi Healthcare Services
Perreault 2009	He Régie de l'assurance maladie du Québec databases.
Corrao 2010	Health service databases of Lombardia
Rasmussen 2007	Ontario Myocardial Infarction Database
Wei 2004	MEMO record-linkage database
Wei 2002	Medicine Monitoring Unit's record linkage database
Esposti 2012	Ealth-Assisted Subjects Database, Medications Prescription Database, Hospital Discharge Database, Mortality Database
Perreault 2009	Régie d'assurance maladie du Québec and Med-Echo databases.
Mazzaglia 2009	Health Search/Thales Database in Italy
Herttua 2013	Statistics Finland Labour Market data; National Drug Reimbursement Register and the Drug Prescription Register kept by the Social Insurance Institution of Finland
Corrao 2011	Health service databases of Lombardy in Italy
Krousel-Wood 2015	The Cohort Study of Medication Adherence in Older Adults in the southeastern Louisiana, USA
Wong 2013	Territory-wide database in Hong Kong
Kim 2016	Korean National Health Insurance
Herttua 2016	The Statistics Finland Labor Market database; National Death Register in Finland; National Drug Reimbursement Register in Finland; the Drug Prescription Register by the Social Insurance Institution of Finland; National Institute for Health and Welfare in Finland
Esposti 2011	Medications Prescription Database maintained by the Local Health Unit of Florence, Italy
Yang 2016	MarketScan Medicaid data
Liu 2009	Taiwan's National Health Insurance reimbursement database
Corrao 2015	healthcare utilization databases of Lombardy

Yang 2017	Medicare fee-for-service beneficiaries aged 66 to 79 years enrolled during 2007–2008
Kim 2016	National Health Insurance Service
Corrao 2017	healthcare utilization databases of Lombardy
Dongen 2019	Care Register for Health Care, the National Institute for Health and Welfare
Lee 2017	National Health Insurance claim data and check-up data
Kim 2018	The homepage of National Health Insurance Sharing Service
Lavie 2021	The Clalit comprehensive health care data warehouse
Lee 2020	National Health Insurance Service National Sample Cohort
Greenland 2020	The Western Australian Data Linkage System - the Hospital Morbidity Data Collection for hospital admissions and Mortality register
Ahrens 2021	German health claims database

CKD, chronic kidney disease

Table S3 The confounders adjusted for the multivariate analysis in each included study

Author	Covariates
Korhonen 2015	Duration of diabetes, type of antidiabetic medication; use of warfarin, platelet inhibitors, digitalis glycosides, nitrates or antihypertensive medication; history of IS, CAD, peripheral arterial disease, congestive heart failure, arrhythmias, moderate/severe hypertension, microangiopathic complications or depression; socioeconomic status, hospital district; type and intensity of the initial statin; and dispensation delay
Bansilal 2016	Demographic characteristics, comorbid conditions, concomitant medication use and copayment, and preventive service use
Karlsson 2017	Sex, age, country of birth, marital status, education level, employment status, profession and individual income, morbidities and diabetes duration, glycated haemoglobin, estimated glomerular filtration rate, BMI, blood lipid levels, blood pressure, microalbuminuria, macroalbuminuria, physical activity and smoking
Rodriguez 2019	Adherence to other cardiac medications, age, statin intensity, sex, race/ethnicity, atherosclerotic cardiovascular disease diagnosis, clinical comorbidities, Council of Teaching Hospitals and Health Systems, and baseline creatinine, vital signs, pulse oximetry, and weight.
Kim 2017	Sex, age, diabetes mellitus, atrial fibrillation, prior myocardial infarction, use of thrombolysis, household income, hospital type, index-stroke year, and adherence to the above medications
Corrao 2017	Index prescription, gender, age and potency of the dispensed statins, hospital admissions for CV events, and the Charlson comorbidity index score, the use of other medicaments
Mathews 2018	Age, gender, race, length of stay, and BMI, indicators of socioeconomic status, additional insurance besides Medicare , comorbidities, STEMI or STEMI equivalent on arrival, transfer-in status, cardiac catheterization within 24 hours of arrival, in-hospital PCI, left ventricular ejection fraction, end-stage renal disease, baseline hemoglobin, in-hospital HF complication, in-hospital major bleeding, and any blood transfusion, hospital geographic region, hospital type, teaching hospital, and hospital bed size.
Yeo 2020	Age, statin prescription at discharge and average time between encounters
Rannanheimo 2015	Sex, Age, hospital district, taxable income, level of education, socioeconomic group, marital status, family type, type of main activity, dysfunction of lipid metabolism, diabetes, hypertension, type of initial statin, intensity of initial statin therapy, dispensation delay, year of statin initiation, antithrombotic agents, diuretics, beta-blocking agents, calcium channel blockers, agents acting on the renin-angiotensin system, number of concurrent CV medications, Parkinson disease, rheumatoid arthritis, certain diseases of the nervous system, depression, antidepressants, respiratory diseases, renal impairments, obesity, diabetic retinopathy, polyneuropathy, sleep apnea, psoriasis, alcoholism/narcomania, nonsteroidal anti-inflammatory drugs, corticosteroids for systemic use, number of concurrent medications, medication costs, and number of hospitals days
Hickson 2019	Sociodemographics, baseline clinical conditions and medication use, whether the patient was a new user of statins, index hospitalization events, postdischarge clinical events and medication use, and changes in statin doses.
Chen 2019	Age, gender, onset year, pre-morbid risk factors, stroke subtype, CCI; and brain imaging, respiratory disease/infections, neurosurgery, rehabilitation use, admission department, hospital level, geographic region, and use of NSAID
Ye 2013	Age, sex, region, comorbidities, and concomitant drugs
Molnar 2016	Age, sex, race/ethnicity, marital status and ZIP code, CCI and presence of diabetes, congestiveheart failure, cardiovascular/cerebrovascular disease, presence of depression, presence of anxiety and type of vascular access
Chen 2016	Age, gender, Charlson index score, diabetes mellitus, anticoagulant agents, ACEI/ARB, calcium channel blockers, diuretics
Phan 2019	Demographics, comorbidities, and exposure to other cardiac medications

Shalev 2012	Age, gender, residential socioeconomic level, diabetes mellitus, hypertension, use of health services, and median baseline lipids levels.
Pittman 2011	Age, gender, coronary artery disease, diabetes, hypertension, stroke, peripheral arterial disease, congestive heart failure, depression, year 1 hospitalization, year 1 emergency department visits, and number of medications in year 1.
Shalev 2009	Age and sex, marital status, nationality, socioeconomic status, years of living in Israel, residence area, chronic condition, visits to primary care physician during the year before the index date, number of hospitalizations during the year before the index date, cancer, diabetes mellitus, and use of antihypertensives and diuretics. mean level of low-density lipoprotein cholesterol during the year before the index date
Perreault 2009	Age, sex, other cardiovascular drugs and previous events
Corrao 2010	Gender, age at entry, statin type for first-line therapy and concomitant use of other drugs
Rasmussen 2007	Age, sex, income, specialty of attending physician, drug therapy, comorbidity at index AMI, readmissions within 1 y after discharge, Observation time, Deaths in observation period
Wei 2004	Age, sex, social deprivation, prior OAD, cardiovascular disease, diabetes mellitus, PVD, prior beta-blocker use, prior use of calcium blockers, angiotensin-converting enzyme inhibitors, alphablockers, antihypertensive drugs, thiazide diuretic, loop diuretic, nitrates, antiplatelet drugs, lipid-lowering and steroid prescriptions
Wei 2002	Age, sex, socioeconomic deprivation, serum cholesterol concentration, diabetes mellitus, cardiovascular drug use, and other hospitalisations.
Esposti 2012	Age, sex, medications before the enrollment date, cardiovascular risk at baseline, and statin adherence level
Perreault 2009	Sex and social assistance, occurrence of coronary artery disease, chronic heart failure, peripheral artery disease, or other cardiovascular disease conditions, diabetes and hypertension, the use of antidiabetic or antihypertensive agents , chronic disease score.
Mazzaglia 2009	Age, sex, use of antithrombotics, concurrent medications, and comorbidities, prior hospitalization, and the number of AHM
Herttua 2013	Age, sex, length of AHM, education, income, comorbidity, and a history of cancer
Corrao 2011	Sex, age, the number of AHM, comorbidity, and drugs prescribed for heart failure or coronary heart disease
Krousel-Wood 2015	Age, sex, race, marital status, education, comorbidities, the number of AHM, BMI, and lifestyle behaviors
Wong 2013	Age, sex, public service, and the class of first AHM
Kim 2016	Age, sex, income, residential regions, comorbidities, and the number of AHM
Herttua 2016	Age, sex, education, comorbidity, and a history of cancer
Esposti 2011	Age, sex, comorbidities, and use of antidiabetic agents, lipid-lowering agents, cardiac therapy, and antiplatelet agents
Yang 2016	Age, sex, race, previous CVD, and comorbidities
Liu 2009	Age, sex, the number of AHM, and comorbidities
Corrao 2015	The antihypertensive drug class, use of statins and antidiabetic and antidepressant agents, and the CCI, the number of antihypertensive drug classes, the switching between antihypertensive drugs, the addition of other cardiovascular drugs to the antihypertensive treatment regimen, and the number of prescribing physicians
Yang 2017	Age, sex, race/ethnicity, urban-rural residence, statins use, number of antihypertensive medications, status of low-income subsidy, out-of-pocket medications payment/day, CCI, and Alzheimer's disease, asthma, chronic kidney disease, chronic obstructive pulmonary disease and bronchiectasis, depression, diabetes mellitus, hyperlipidemia, lung or colorectal cancer, and rheumatoid arthritis/osteoarthritis.
Kim 2016	Gender, age, the type of health insurance coverage, the main types of medical institutions, the location of medical institutions, CCI, past history of

	hospitalization and diabetes mellitus , the number of anti-hypertensive drugs, and the number of medical institutions for the 1st one year .
Corrao 2017	Antihypertensive treatment strategy; drug class; the use of antiarrhythmic, antiplatelet, oral anticoagulant, lipid-lowering, antidiabetic and antidepressant agents; the CCI
Dongen 2019	Age, sex, types 1 and 2 diabetes mellitus, cigarette smoking, heart failure, pre-existing hypertension, dyslipidemia, heavy drinking, contraceptive pill use, modified TOAST classification, NIHSS at admission and prior use of antihypertensive medication.
Lee 2017	Age, sex, income, region, CCI, metabolic syndrome, family history of stroke, smoking, regular exercise, type of hypertension, duration of hypertension and year.
Kim 2018	Type of hemorrhagic stroke , sex, age, diabetes mellitus, prior myocardial infarction, household income, length of hospital stay, and year of index hemorrhagic stroke
Lavie 2021	Age, sex, socioeconomic status, immigration status, systolic and diastolic blood pressure, smoking status, BMI level, blood glucose concentration (mg/dl), total cholesterol, LDL cholesterol, HDL cholesterol, triglycerides, CCI and medication use: calcium channel blockers, use of beta-blockers, thiazides, oral steroids, oral contraceptives, antipsychotics, phenytoin, thyroxine, and fibrates.
Lee 2020	NR
Greenland 2020	age, sex, accessibility/remoteness, history of: hypertension, heart failure, atrial fibrillation, diabetes, chronic obstructive pulmonary disease, chronic kidney disease, stroke, peripheral vascular disease, coronary heart disease, coronary artery revascularization procedure, coronary heart disease admissions with or without coronary artery revascularisation procedure in the one-year landmark period and concomitant cardioprotective drugs.
Ahrens 2021	age, sex, history of chronic CVD conditions (any of the following: carotid stenosis, peripheral artery disease, abdominal aortic aneurysm, or other cardiac ischaemia), type 2 diabetes, chronic kidney disease stage 4–5, atrial fibrillation, anti-thrombotic medication use, hypertension, CCI and acute cardiovascular hospitalisation in the past year.

AHM, antihypertensive medication; CVD, cardiovascular disease; CCI, Charlson comorbidity Index; BMI, body mass index; SBP, systolic blood pressure; DBP, diastolic blood pressure; HIV, human immunodeficiency virus; AIDS, acquired immune deficiency syndrome; CHF, congestive heart failure; PAD, peripheral artery diseases; CAD, coronary artery disease; TIA, transient ischemic attacks; HF, heart failure; CV, cardiovascular; BP, blood pressure; CKD, chronic kidney disease; ASCVD, atherosclerotic cardiovascular disease; CHD, coronary heart disease ;AMI, myocardial infarction; NSAID, nonsteroidal anti-inflammatory drug; PCI, percutaneous coronary intervention; NR, not reported.

Table S4 Quality assessment of the included studies*

Reference	Is the exposed cohort representative?	Selection of the non-exposed cohort	Ascertainment of exposure	Demonstration that outcome of interest was not present at start of study	Comparability of important factors†	Assessment of outcome	Follow up period	Adequacy of follow up of cohorts	Score
Korhonen 2015	1	1	1	1	2	1	1	1	9
Bansilal 2016	1	1	1	1	1	1	0	1	7
Karlsson 2017	1	1	1	1	2	1	0	1	8
Rodriguez 2019	0	1	1	1	2	1	0	1	7
Kim 2017	1	1	1	1	1	1	1	1	8
Corrao 2017	1	1	1	1	2	1	0	1	8
Mathews 2018	1	1	1	1	2	0	0	1	7
Yeo 2020	1	1	1	1	2	1	0	0	7
Rannanheimo 2015	1	1	1	1	2	1	0	1	8
Hickson 2019	1	1	1	1	2	1	0	1	8
Chen 2019	1	1	1	1	2	1	1	1	9
Ye 2013	1	1	1	1	1	1	0	1	7
Molnar 2016	1	1	1	1	2	1	0	1	8
Chen 2016	1	1	1	1	2	1	0	1	8
Phan 2019	1	1	1	1	2	1	0	1	8
Shalev 2012	1	1	1	1	2	1	1	1	9
Pittman 2011	1	1	1	1	2	1	0	1	8
Shalev 2009	1	1	1	1	2	0	1	1	8
Perreault 2009	1	1	1	1	2	1	1	1	9
Corrao 2010	1	1	1	1	2	1	0	1	8
Rasmussen 2007	1	1	1	1	2	1	1	1	9
Wei 2004	1	1	1	1	2	1	1	1	9
Wei 2002	1	1	1	1	1	1	1	1	8
Esposti 2012	1	1	1	1	1	1	1	1	8

Perreault 2009	1	1	1	1	2	1	1	1	9
Corrao 2015	1	1	0	1	2	1	1	0	7
Yang 2017	1	1	1	1	1	1	1	1	8
Lee 2017	1	1	1	1	2	1	1	1	9
Kim 2018	1	1	1	1	2	1	1	1	9
Krousel-Wood 2018	1	1	0	1	2	1	1	1	8
Corrao 2017	0	1	1	1	2	1	1	1	8
Kim 2016	1	1	1	1	2	1	1	1	9
Dongen 2019	1	0	1	1	2	1	1	0	7
Mazzaglia 2009	1	1	1	1	1	1	1	1	8
Herttua 2013	1	1	1	1	2	1	1	1	9
Corrao 2011	1	1	1	1	1	1	1	1	8
Krousel-Wood 2015	1	1	1	1	2	1	1	1	9
Wong 2013	1	1	1	1	0	1	1	1	7
Esposti 2011	1	1	1	1	1	1	1	1	8
Yang 2016	1	1	1	1	1	0	1	1	8
Liu 2009	1	1	1	1	2	1	1	1	9
Herttua 2016	1	1	1	1	2	1	1	1	9
Lavie 2021	0	1	1	1	0	1	1	1	6
Lee 2020	0	1	0	1	1	0	1	1	5
Greenland 2020	0	1	1	1	0	1	1	1	6
Ahrens 2021	1	1	1	1	0	0	1	1	6

*Newcastle-Ottawa Scale was used to assess the study quality in this meta-analysis. The full score was 9 stars, and the high-quality study was defined as a study with 8 awarded stars.

† A maximum of two stars could

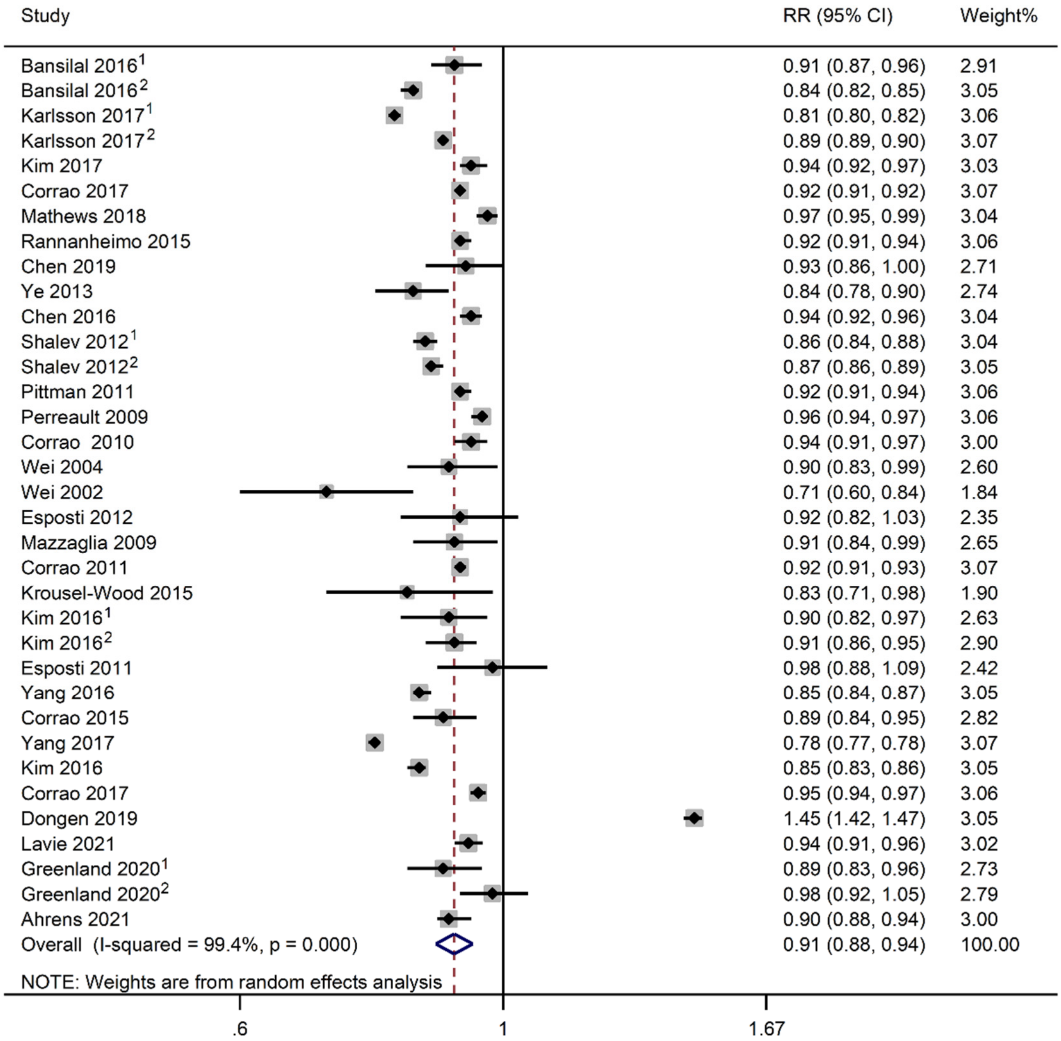

Figure S1 Forest plot of study-specific relative risk statistics for total cardiovascular diseases per 20% increment of cardiovascular medications adherence

Bansilal 2016[1]: Post-MI cohorts, Bansilal 2016[2]: atherosclerosis cohorts, Karlsson 2017[1]: primary prevention, Karlsson 2017[2]: secondary prevention, Shalev 2012[1]: women, Shalev 2012[2]: men, Kim 2016[1]: acute myocardial infarction, Kim 2016[2]: ischemic heart disease.

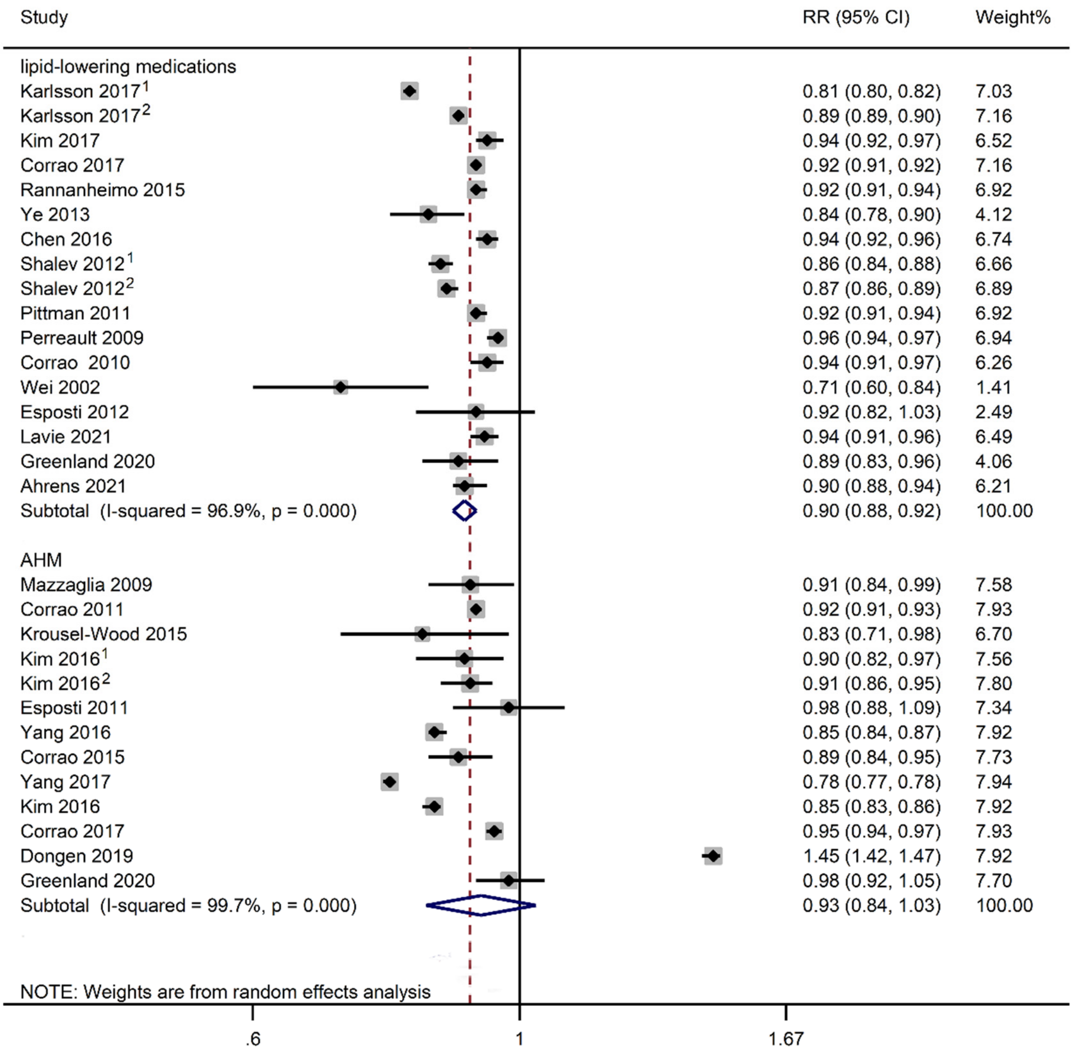

Figure S2 Forest plot of study-specific relative risk statistics for total cardiovascular diseases per 20% increment of antihypertensive medication adherence (AHM) and lipid-lowering medications adherence Kim 2016[1]: acute myocardial infarction, Kim 2016[2]: ischemic heart disease, Karlsson 2017[1]: primary prevention, Karlsson 2017[2]: secondary prevention, Shalev 2012[1]: women, Shalev 2012[2]: men

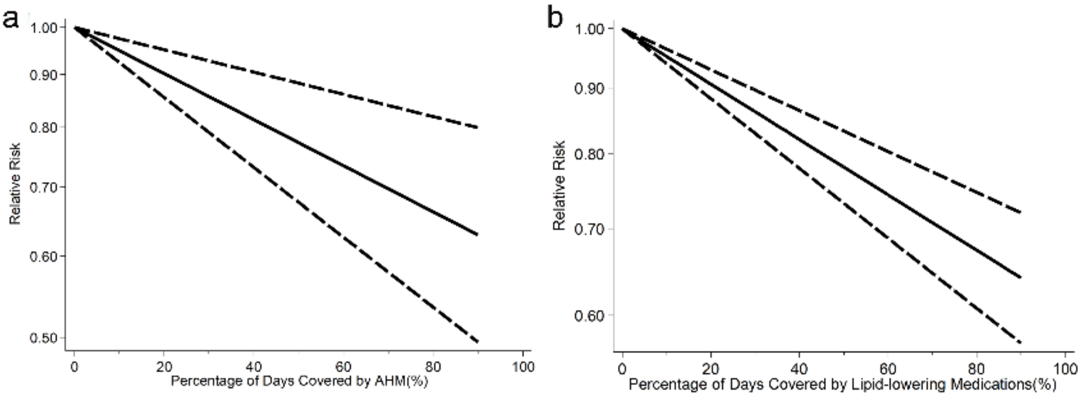

Figure S3 (a) Pooled dose-response analysis of antihypertensive medication adherence and total Cardiovascular Diseases risk (solid line). (**b)** Pooled dose-response analysis of lipid-lowering medication adherence and total Cardiovascular Diseases risk (solid line).
Dashed lines represent the 95% CI.

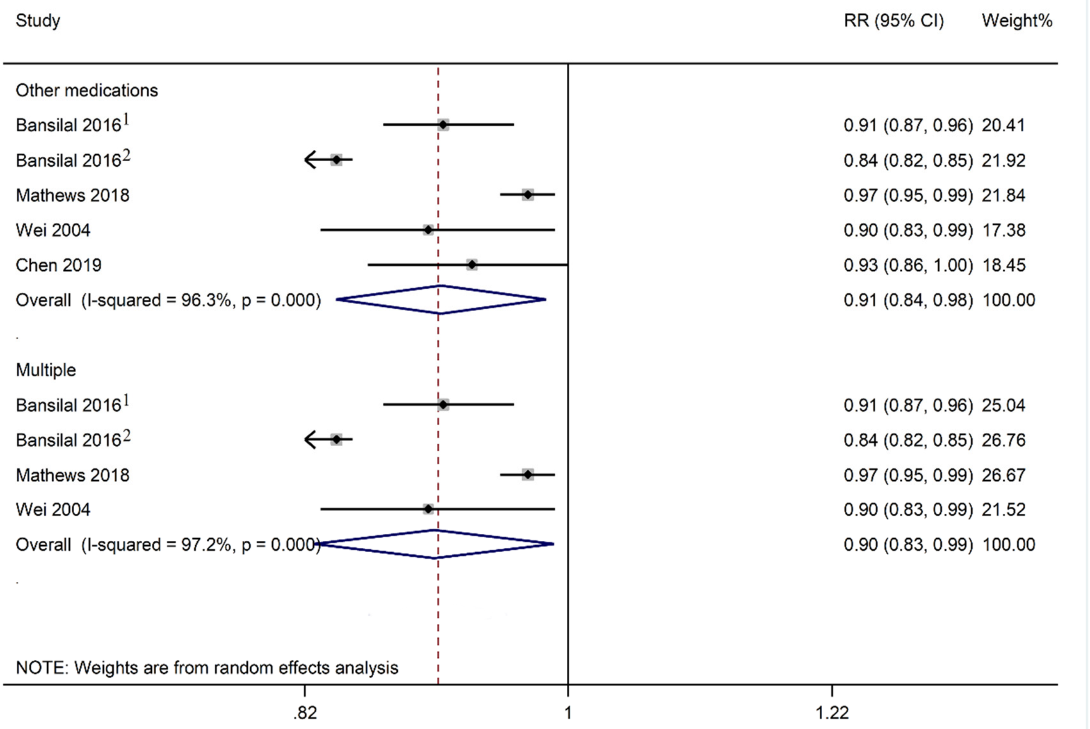

Figure S4 Forest plot of study-specific relative risk statistics for total cardiovascular diseases per 20% increment of multiple medication and other medication（multiple and antithrombotic）adherence.
Bansilal 2016[1]: Post-MI cohorts, Bansilal 2016[2]: atherosclerosis cohorts

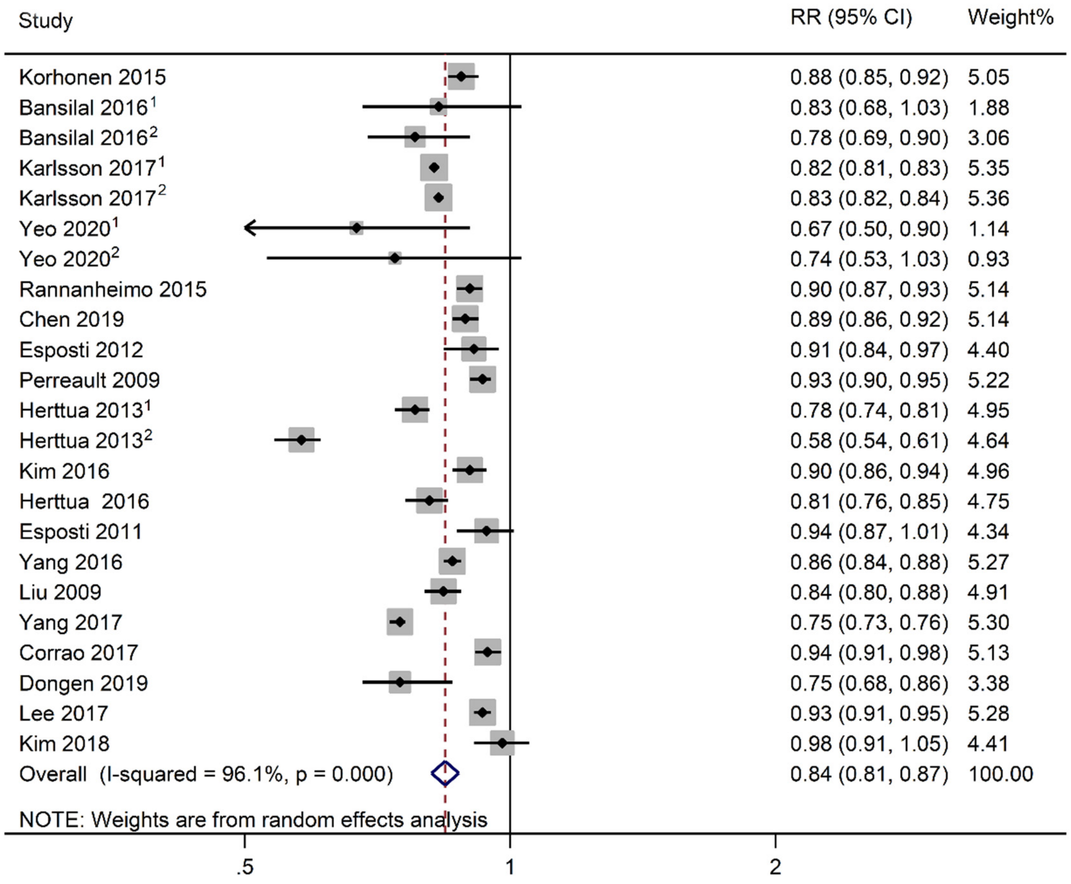

Figure S5 Forest plot of study-specific relative risk statistics for stroke per 20% increment of medications adherence.

Bansilal 2016[1]: Post-MI cohorts, Bansilal 2016[2]: atherosclerosis cohorts, Karlsson 2017[1]: primary prevention, Karlsson 2017[2]: secondary prevention, Yeo 2020: antithrombotics, Yeo 2020: stains, Herttua 2013[1]: non-fatal stroke, Herttua 2013[2]: fatal stroke

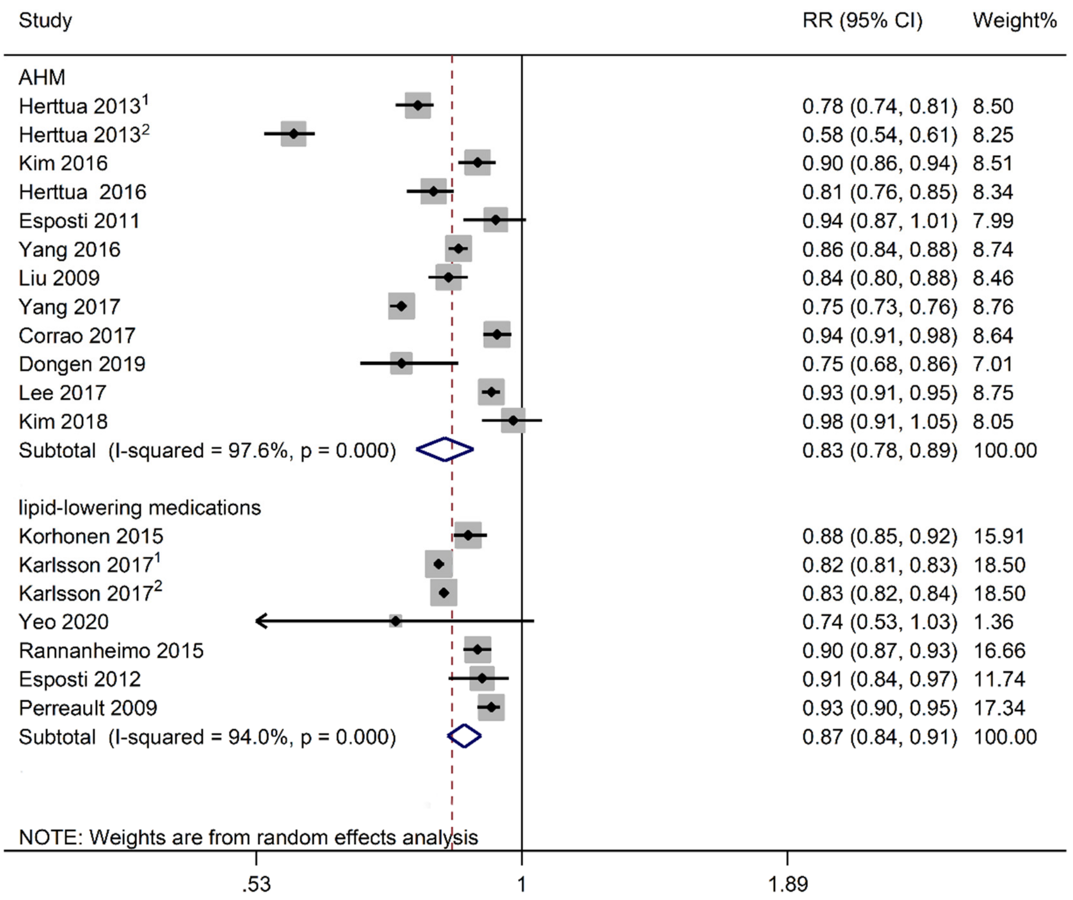

Figure S6 Forest plot of study-specific relative risk statistics for stroke per 20% increment of antihypertensive medication adherence (AHM) and lipid-lowering medications adherence.

Herttua 2013[1]: non-fatal stroke, Herttua 2013[2]: fatal stroke; Karlsson 2017[1]: primary prevention Karlsson 2017[2]: secondary prevention

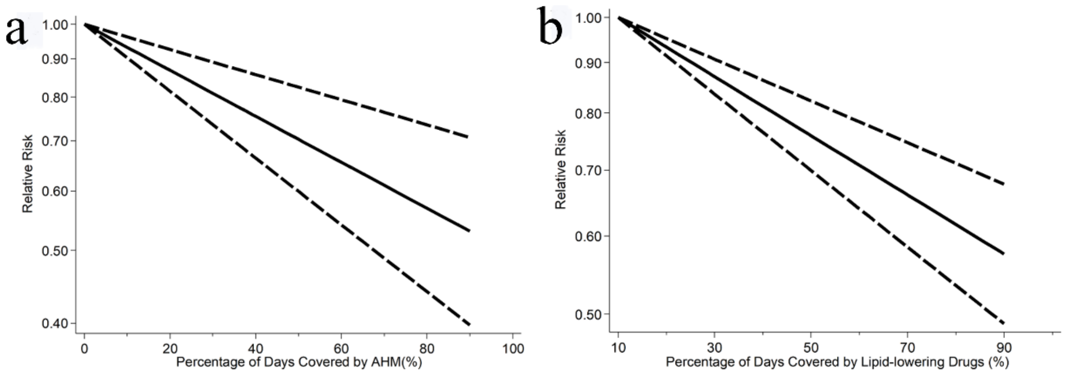

Figure S7 (a) Pooled dose-response analysis of antihypertensive medication adherence and stroke risk (solid line). (**b**) Pooled dose-response analysis of lipid-lowering medication adherence and stroke risk (solid line). Dashed lines represent the 95% CI.

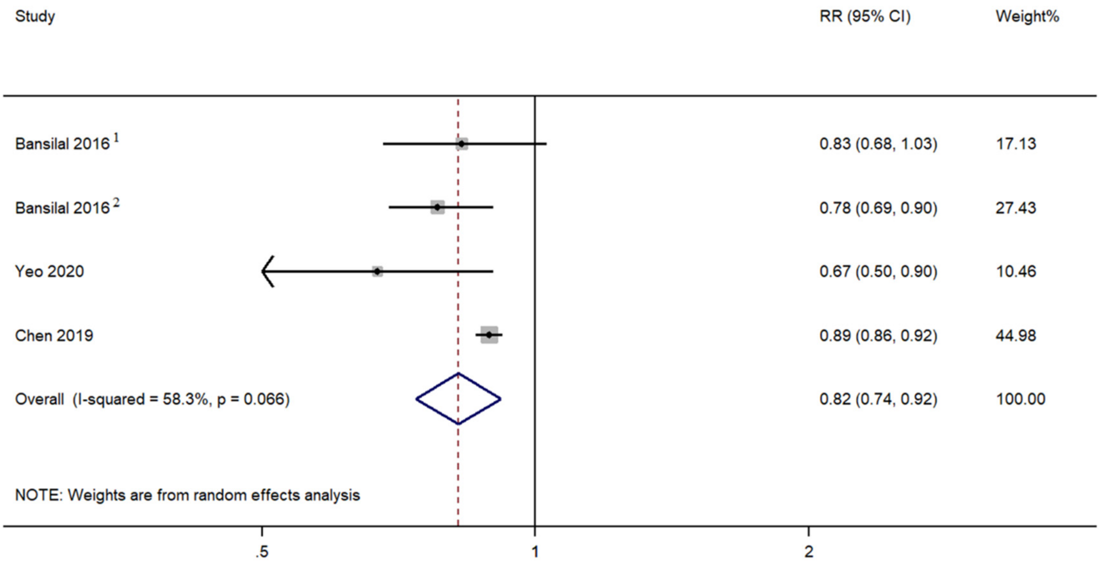

Figure S8 Forest plot of study-specific relative risk statistics for stroke per 20% increment of other medication（multiple and antithrombotic）adherence.

Bansilal 2016[1]: Post-MI cohorts, Bansilal 2016[2]: atherosclerosis cohorts

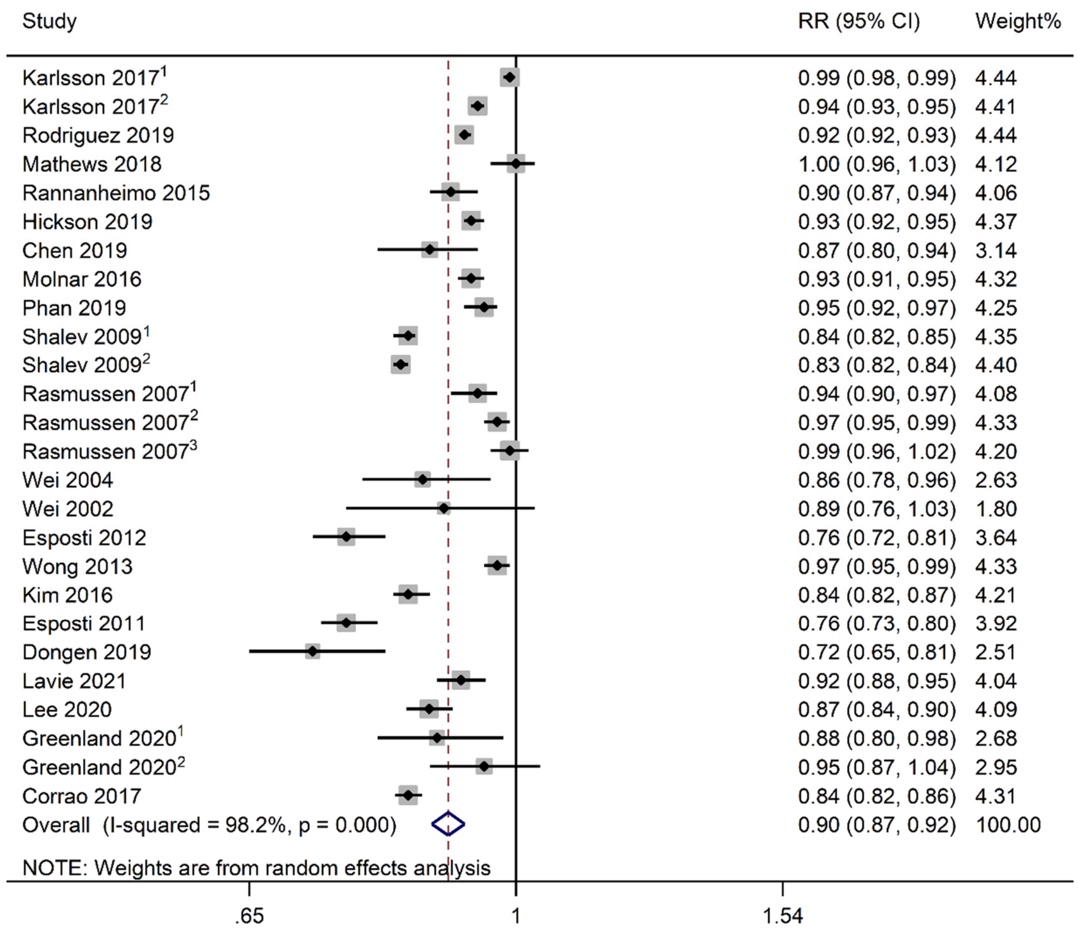

Figure S9 Forest plot of study-specific relative risk statistics for all-cause mortality per 20% increment of medications adherence.

Karlsson 2017[1]: primary prevention Karlsson 2017[2]: secondary prevention, Shalev 2009[1]: primary prevention, Shalev 2009[2]: secondary prevention, Rasmussen 2007[1]: statins, Rasmussen 2007[2]: beta-blockers, Rasmussen 2007[3]: calcium channel blockers.

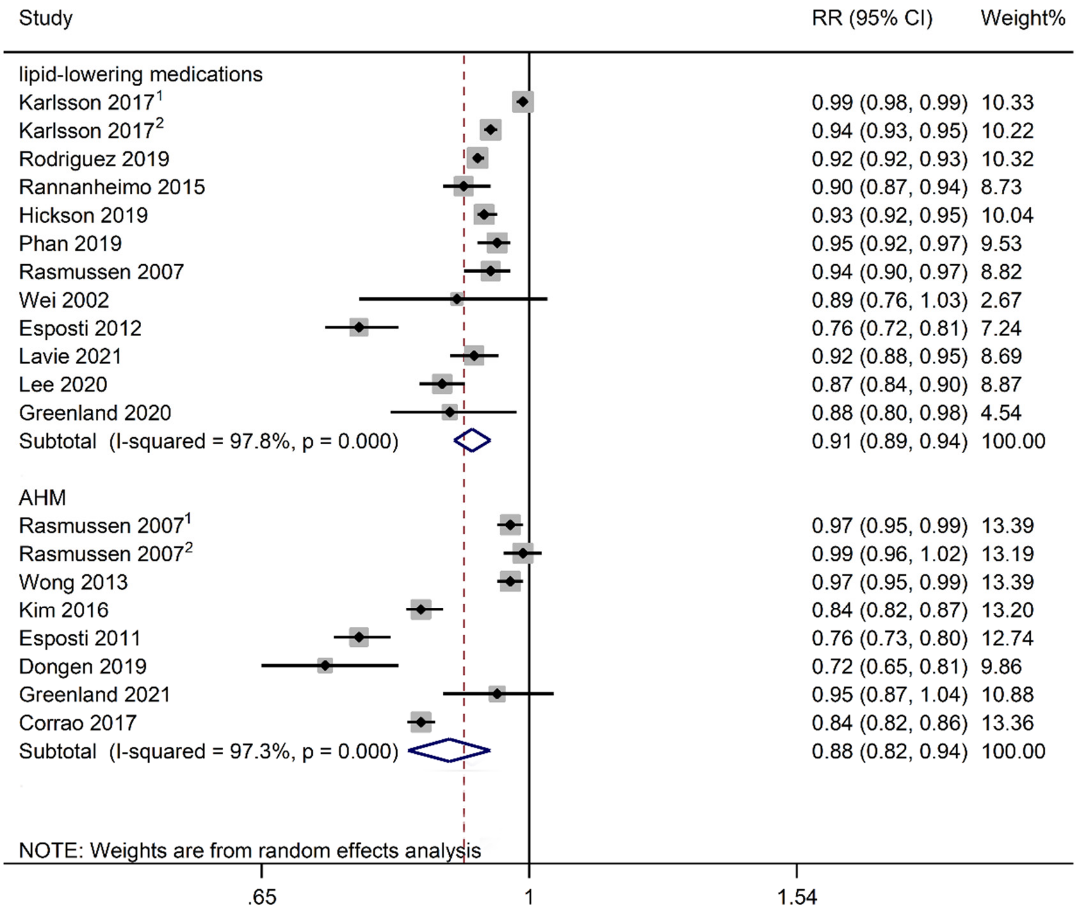

Figure S10 Forest plot of study-specific relative risk statistics for all-cause mortality per 20% increment of antihypertensive medication adherence (AHM) and lipid-lowering medications adherence. Rasmussen 2007[1]: beta-blockers, Rasmussen 2007[2]: calcium channel blockers; Karlsson 2017[1]: primary prevention; Karlsson 2017[2]: secondary prevention.

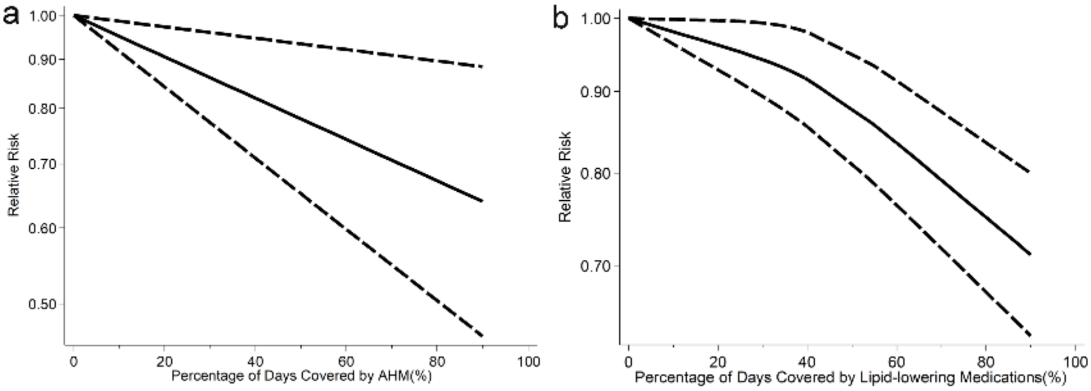

Figure S11 (**a**) Pooled dose-response analysis of antihypertensive medication adherence and all-cause mortality risk (solid line). (**b**) Pooled dose-response analysis of lipid-lowering medication adherence and all-cause mortality risk (solid line). Dashed lines represent the 95% CI.

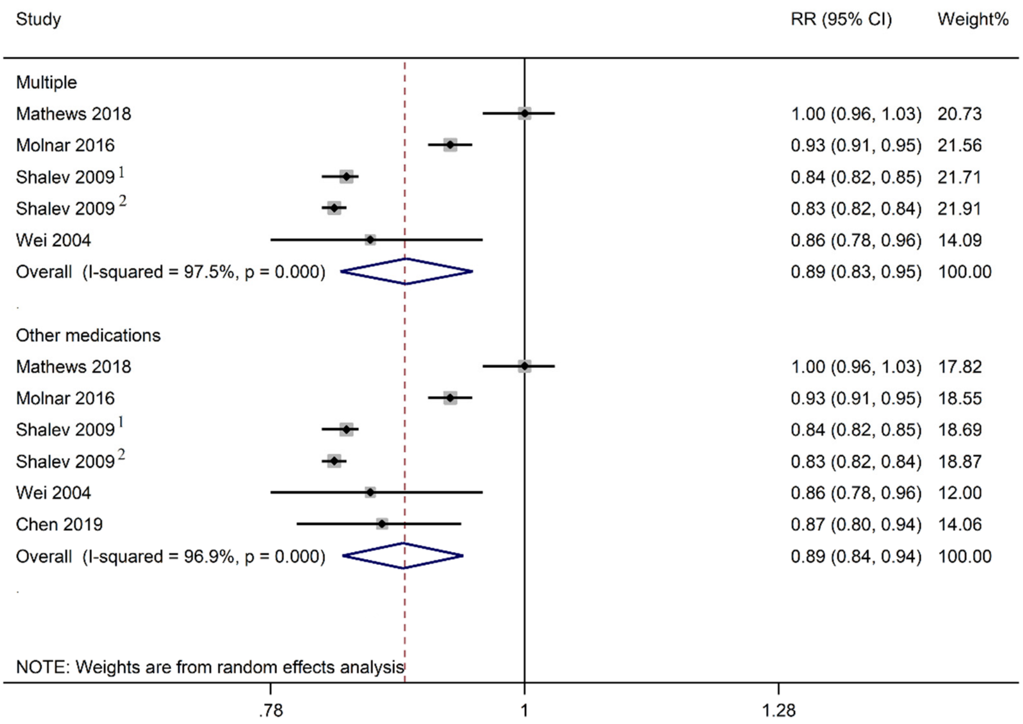

Figure S12 Forest plot of study-specific relative risk statistics for total cardiovascular diseases per 20% increment of multiple medication and other medication (multiple and antithrombotic) adherence. Shalev 2009[1]: primary prevention, Shalev 2009[2]: secondary prevention

Subgroups	No. of studies	RR	95%*CI*	*I-square* (%)	*P* Value
**All studies**	35	0.91	(0.88-0.94)	99.4	<0.001
**Age**					
<65	18	0.92	(0.87-0.97)	99.5	<0.001
≥65	14	0.9	(0.84-0.95)	99.3	<0.001
**Follow-up**					
<5	24	0.91	(0.88-0.93)	96.3	<0.001
≥5	10	0.92	(0.82-1.04)	99.8	<0.001
**Location**					
North America	9	0.88	(0.82-0.94)	99.3	<0.001
Europe	18	0.93	(0.88-0.98)	99.5	<0.001
Asia	8	0.91	(0.88-0.94)	89.1	<0.001
**Strategies for the assessment of adherence**					
PDC	23	0.93	(0.88-0.98)	99.6	<0.001
MPR	10	0.88	(0.85-0.91)	97.8	<0.001
CMA	2	0.91	(0.88-0.94)	0	0.824
**Study quality**					
<8	10	0.95	(0.82-1.10)	99.6	<0.001
≥8	25	0.89	(0.87-0.92)	98.9	<0.001
**Prevention level**					
PP	22	0.89	(0.86-0.92)	99.1	<0.001
SP	13	0.93	(0.83-1.05)	99.5	<0.001
**Adjusted for other co-medications**					
Yes	23	0.9	(0.87-0.93)	99.0	<0.001
No	12	0.93	(0.84-1.04)	99.6	<0.001
**Adjusted for previous comorbidity**					
Yes	27	0.89	(0.87-0.92)	98.8	<0.001
No	8	0.97	(0.85-1.11)	99.7	<0.001
**Adjusted for socioeconomic status**					
Yes	10	0.89	(0.86-0.92)	98	<0.001
No	25	0.92	(0.86-0.98)	99.5	<0.001
**Adjusted for the class of medications**					
Yes	5	0.93	(0.91-0.95)	82.0	<0.001
No	30	0.91	(0.87-0.95)	99.4	<0.001
**Adjusted for the number of medications**					
Yes	9	0.88	(0.82-0.94)	99.1	<0.001
No	26	0.92	(0.88-0.96)	99.3	<0.001
**Baseline population**					
Healthy	1	0.94	(0.91-0.97)	-	-
Hypertensive	10	0.89	(0.83-0.95)	99.2	<0.001
Hypercholesterolaemia	8	0.91	(0.89-0.93)	93.1	<0.001
Diabetic	3	0.85	(0.78-0.91)	98.9	<0.001
Known prior CVD	13	0.93	(0.83-1.05)	99.5	<0.001

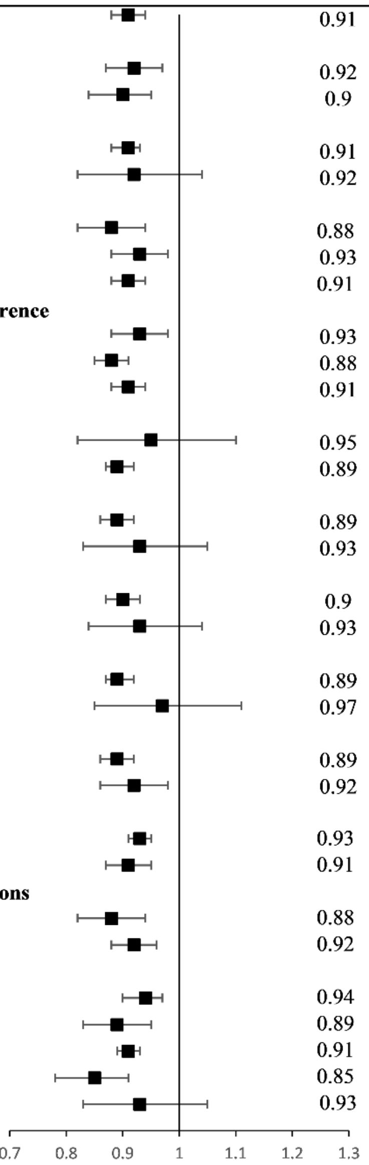

Figure S13 Subgroup analysis of dose response relation of risk of total cardiovascular diseases with cardiovascular medication adherence.

RR: relative risk; CI: confidence interval; PDC: proportion of days covered; MPR: medication possession ratio; CMA: cumulative medication adherence. PP: primary prevention, SP: secondary prevention. CVD: cardiovascular disease. *P* for heterogeneity within each subgroup estimated by the Cochran *Q* test.

Subgroups	No. of studies	RR	95%CI	*I-square* (%)	*P* Value
**All studies**	23	0.85	(0.82-0.88)	95.9	<0.001
**Age**					
<65	13	0.87	(0.84-0.90)	94.0	<0.001
≥65	4	0.87	(0.77-0.99)	98.2	<0.001
**Follow-up**					
<5	13	0.89	(0.86-0.92)	93.2	<0.001
≥5	10	0.78	(0.72-0.85)	97.6	<0.001
**Location**					
North America	4	0.80	(0.72-0.89)	96.1	<0.001
Europe	12	0.84	(0.80-0.87)	96.5	<0.001
Aisa	7	0.90	(0.86-0.94)	77.3	<0.001
**Strategies for the assessment of adherence**					
PDC	16	0.82	(0.77-0.88)	96.1	<0.001
MPR	5	0.87	(0.83-0.92)	97.5	<0.001
Other	2	0.87	(0.81-0.93)	76.8	<0.001
**Study quality**					
<8	5	0.76	(0.71-0.82)	0.0	0.808
≥8	18	0.85	(0.82-0.89)	96.9	<0.001
**Prevention level**					
PP	16	0.85	(0.81-0.88)	97.2	<0.001
SP	7	0.83	(0.76-0.91)	74.9	<0.001
**Adjusted for other co-medications**					
Yes	11	0.87	(0.83-0.90)	96.3	<0.001
No	12	0.82	(0.77-0.87)	95.8	<0.001
**Adjusted for previous comorbidity**					
Yes	18	0.84	(0.81-0.87)	96.8	<0.001
No	5	0.83	(0.74-0.95)	80.0	0.001
**Adjusted for socioeconomic status**					
Yes	7	0.82	(0.78-0.87)	97.9	<0.001
No	16	0.85	(0.81-0.90)	94.4	<0.001
**Adjusted for the class of medications**					
Yes	2	0.92	(0.88-0.96)	73.0	0.09
No	21	0.84	(0.81-0.87)	96.0	<0.001
**Adjusted for the number of medications**					
Yes	3	0.83	(0.73-0.93)	96.9	<0.001
No	20	0.85	(0.82-0.88)	95.5	<0.001
**Baseline population**					
Hypertensive	9	0.83	(0.76-0.90)	98.1	<0.001
Hypercholesterolaemi	4	0.89	(0.84-0.94)	84.3	<0.001
Diabetic	3	0.84	(0.82-0.86)	82.9	0.003
Known prior CVD	7	0.83	(0.76-0.91)	74.9	0.001

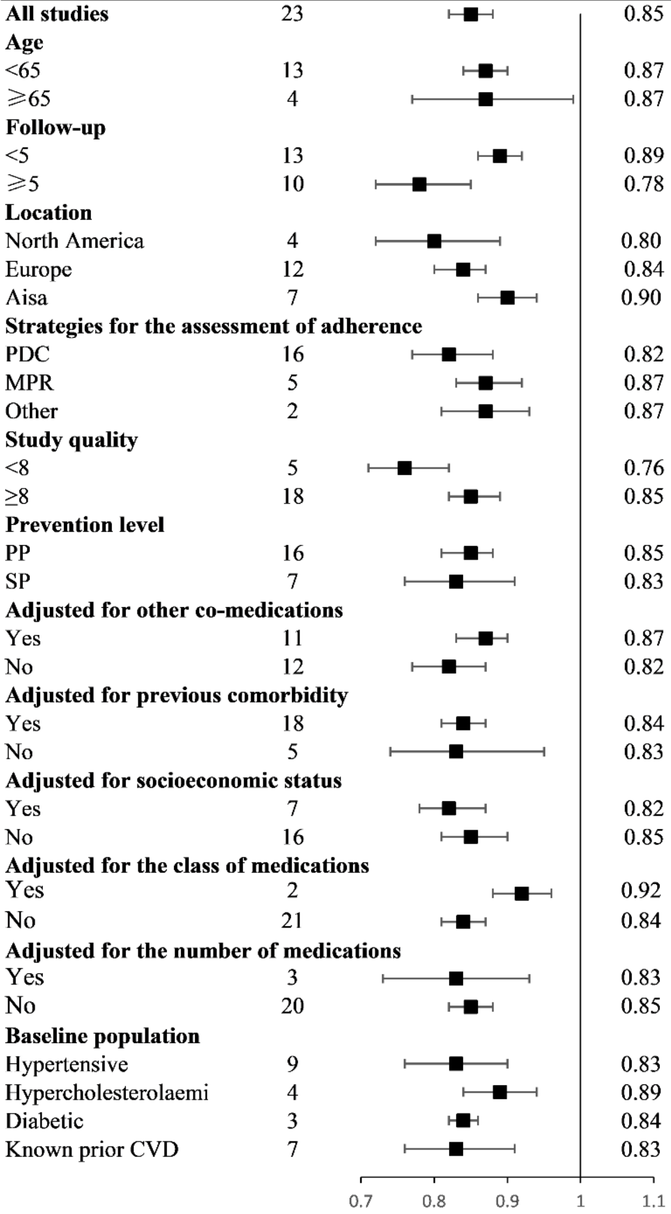

Figure S14 Subgroup analysis of dose response relation of risk of stroke with cardiovascular medication adherence.

RR: relative risk; CI: confidence interval; PDC: proportion of days covered; MPR: medication possession ratio; PMC: proportion of months covered; CMA: cumulative medication adherence. PP: primary prevention, SP: secondary prevention. CVD: cardiovascular disease. *P* for heterogeneity within each subgroup estimated by the Cochran *Q* test.

Subgroups	No. of studies	RR	95%*CI*	*I-square* (%)	*P* Value
**All studies**	26	0.90	(0.87-0.92)	98.2	<0.001
**Age**					
<65	8	0.86	(0.81-0.92)	99.3	<0.001
≥65	13	0.91	(0.88-0.94)	92.8	<0.001
**Follow**					
<5	18	0.91	(0.88-0.94)	96.7	<0.001
≥5	7	0.86	(0.82-0.91)	96.9	<0.001
**Location**					
North America	5	0.94	(0.92-0.96)	85.2	<0.001
Europe	14	0.89	(0.85-0.92)	97.4	<0.001
Aisa	7	0.87	(0.83-0.92)	96.7	<0.001
**Strategies for the assessment of adherence**					
PDC	19	0.89	(0.86-0.92)	96.9	<0.001
MPR	5	0.93	(0.89-0.95)	99.0	<0.001
Other	2	0.88	(0.80-0.98)	96.6	<0.001
**Study quality**					
<8	8	0.92	(0.88-0.95)	90.7	<0.001
≥8	18	0.89	(0.86-0.92)	98.7	<0.001
**Adjusted for other co-medications**					
Yes	16	0.91	(0.88-0.93)	97.0	<0.001
No	10	0.88	(0.85-0.92)	97.9	<0.001
**Adjusted for previous comorbidity**					
Yes	19	0.92	(0.89-0.94)	97.4	<0.001
No	6	0.84	(0.78-0.89)	97.5	<0.001
**Adjusted for socioeconomic status**					
Yes	13	0.93	(0.89-0.97)	98.7	<0.001
No	13	0.87	(0.84-0.90)	95.4	<0.001
**Adjusted for the class of medications**					
Yes	3	0.90	(0.82-0.99)	97.7	0.001
No	23	0.90	(0.87-0.92)	98.3	<0.001
**Adjusted for the number of medications**					
Yes	1	0.84	(0.82-0.87)	-	-
No	25	0.90	(0.88-0.92)	98.2	<0.001
**Baseline population**					
Hypertensive	4	0.85	(0.77-0.94)	98.0	<0.001
Hypercholesterolaemia	6	0.85	(0.82-0.88)	89.7	<0.001
Diabetic	2	0.96	(0.92-1.02)	98.7	<0.001
Known prior CVD	14	0.93	(0.92-0.95)	85.9	<0.001

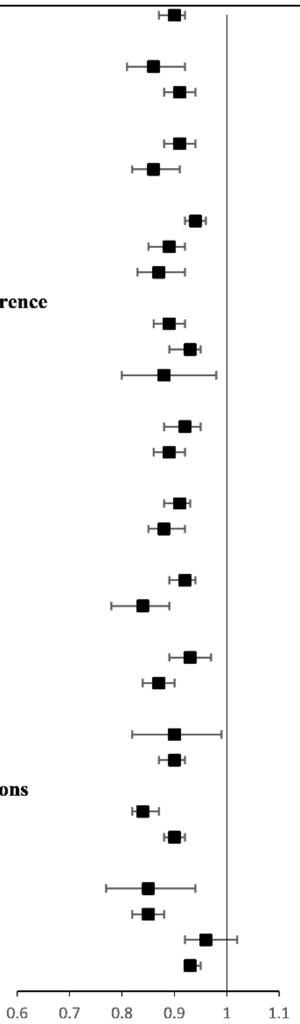

Figure S15 Subgroup analysis of dose response relation of risk of all-cause mortality with cardiovascular medication adherence.

RR: relative risk; CI: confidence interval; PDC: proportion of days covered; MPR: medication possession ratio; PMC: proportion of months covered; CMA: cumulative medication adherence. CVD: cardiovascular disease. *P* for heterogeneity within each subgroup estimated by the Cochran *Q* test.

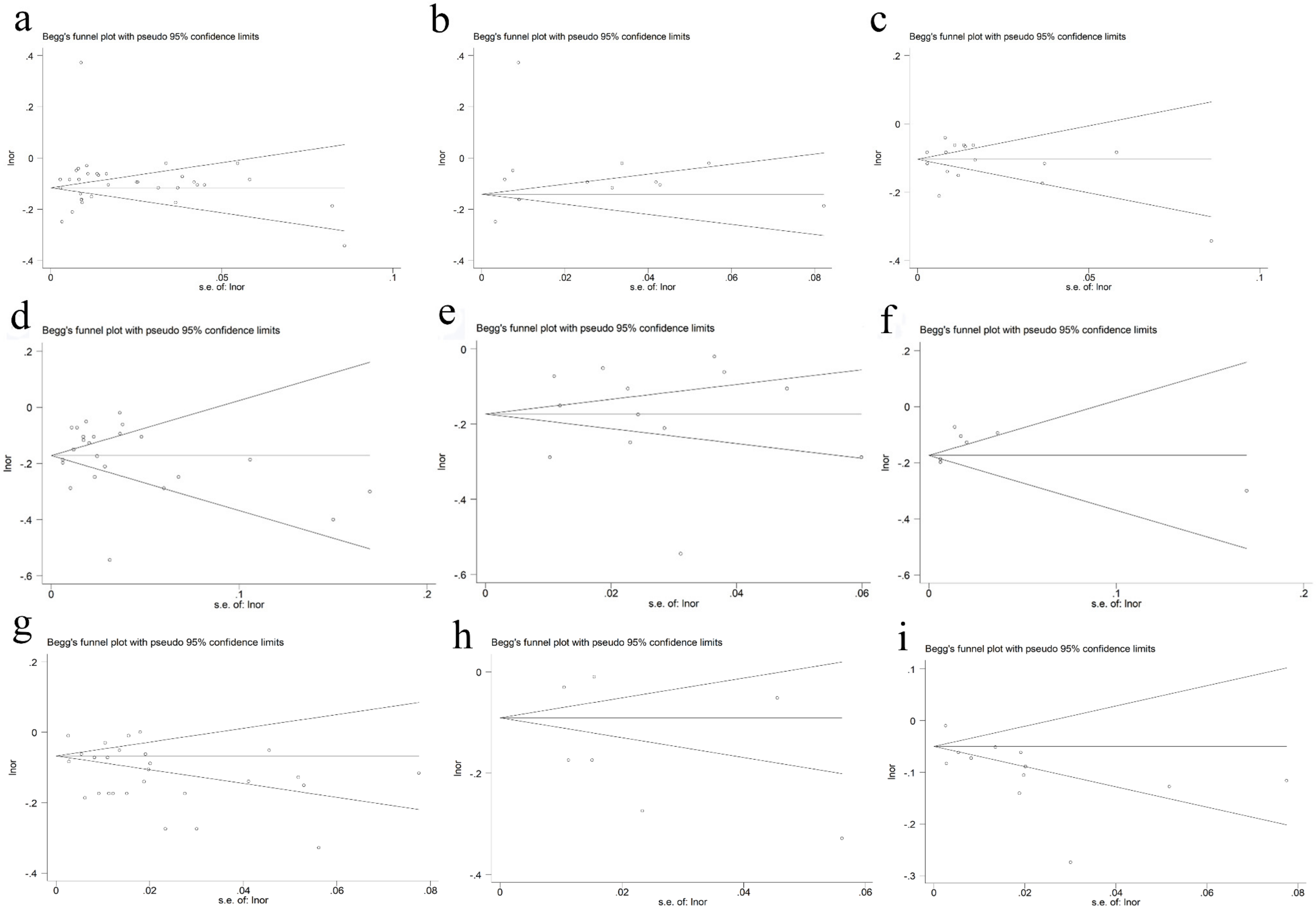
a
Begg's funnel plot with pseudo 95% confidence limits
lnor
s.e. of: lnor
b
Begg's funnel plot with pseudo 95% confidence limits
lnor
s.e. of: lnor
c
Begg's funnel plot with pseudo 95% confidence limits
lnor
s.e. of: lnor
d
Begg's funnel plot with pseudo 95% confidence limits
lnor
s.e. of: lnor
e
Begg's funnel plot with pseudo 95% confidence limits
lnor
s.e. of: lnor
f
Begg's funnel plot with pseudo 95% confidence limits
lnor
s.e. of: lnor
g
Begg's funnel plot with pseudo 95% confidence limits
lnor
s.e. of: lnor
h
Begg's funnel plot with pseudo 95% confidence limits
lnor
s.e. of: lnor
i
Begg's funnel plot with pseudo 95% confidence limits
lnor
s.e. of: lnor

Figure S16 (a) Publication bias test for the association between cardiovascular medications adherence and total cardiovascular diseases risk. (**b)** Publication bias test for the association between antihypertensive medications adherence and total cardiovascular diseases risk. (**c)** Publication bias test for the association between lipid-lowering medications adherence and total cardiovascular diseases risk. (**d)** Publication bias test for the association between cardiovascular medications adherence and stroke risk. (**e)** Publication bias test for the association between antihypertensive medications adherence and stroke risk. (**f)** Publication bias test for the association between lipid-lowering medications adherence and stroke risk. (**g)** Publication bias test for the association between cardiovascular medications adherence and all-cause mortality risk. (**h)** Publication bias test for the association between antihypertensive medications adherence and all-cause mortality risk. (**i)** Publication bias test for the association between lipid-lowering medications adherence and all-cause mortality risk. Begg's test, $p > |z| = 0.005$ (continuity corrected)

S1. Search strategy

PubMed database:

#1	“medication adherence” [MeSH Terms] OR “medication compliance” [MeSH Terms] OR “medication persistence” [MeSH Terms] OR “medication adherence” [All Fields] OR "medication compliance" [All Fields] OR "medication persistence"[All Fields] OR “adherence”[All Fields] OR “persistence”[All Fields]
#2	"antihypertensive agents"[MeSH Terms] OR "hydroxymethylglutaryl-coa reductase inhibitors"[MeSH Terms] OR "aspirin"[MeSH Terms] OR "clopidogrel"[MeSH Terms] OR "adrenergic beta-antagonists"[MeSH Terms] OR "hypoglycemic agents"[MeSH Terms]
#3	“Cardiovascular Diseases” [MeSH Terms] OR “Coronary Artery Disease” [MeSH Terms] OR “Atherosclerosis” [MeSH Terms] OR “Coronary Disease” [MeSH Terms] OR “Myocardial Infarction” [MeSH Terms] OR “Myocardial Ischemia” [MeSH Terms] OR “Stroke” [MeSH Terms] OR “Cerebrovascular” [All Fields] OR “Mortality” [MeSH Terms] OR “All cause mortality” [All Fields]
#4	#1 AND #2 AND #3

Web of Science database:

#1	TI= (medication adherence OR medication compliance OR medication persistence)
#2	TS= (medication adherence OR medication compliance OR medication persistence OR adherence OR persistence)
#3	#1 OR #2
#4	TI= (antihypertensive agents OR hydroxymethylglutaryl-coa reductase inhibitors OR aspirin OR clopidogrel OR adrenergic beta-antagonists OR hypoglycemic agents)
#5	TI= (Cardiovascular Diseases OR Coronary Artery Disease OR Atherosclerosis OR Coronary Disease OR Myocardial Infarction OR Myocardial Ischemia OR Stroke OR Cerebrovascular)
#7	TI= (Mortality)
#8	TS= (All-cause mortality)
#9	#6 OR #7 OR #8
#10	#3 AND #4 AND #9

EMBASE database：

(('medication adherence'/exp OR 'medication adherence':ab,ti) OR ('medication compliance'/exp OR 'medication compliance':ab,ti) OR 'medication persistence':ab,ti OR ('adherence'/exp OR adherence:ab,ti) OR ('persistence'/exp OR persistence:ab,ti)) AND (('antihypertensive agent'/exp OR 'antihypertensive agent':ab,ti) OR ('hydroxymethylglutaryl-coa reductase inhibitors'/exp OR 'hydroxymethylglutaryl-coa reductase inhibitors':ab,ti) OR ('aspirin'/exp OR aspirin:ab,ti) OR ('clopidogrel'/exp OR 'clopidogrel':ab,ti) OR ('adrenergic beta-antagonists'/exp OR 'adrenergic beta-antagonists':ab,ti) OR ('antidiabetic agent'/exp OR 'antidiabetic agent':ab,ti)) AND ((('cardiovascular diseases'/exp OR 'cardiovascular diseases':ab,ti) OR ('coronary artery disease'/exp OR 'coronary artery disease':ab,ti) OR ('atherosclerosis'/exp OR atherosclerosis:ab,ti) OR ('coronary disease'/exp OR 'coronary disease':ab,ti) OR ('myocardial infarction'/exp OR 'myocardial infarction':ab,ti) OR ('myocardial ischemia'/exp OR 'myocardial ischemia':ab,ti) OR ('stroke'/exp OR stroke:ab,ti) OR ('cerebrovascular disease'/exp OR 'cerebrovascular disease':ab,ti)) OR (('mortality'/exp OR 'mortality':ab,ti) OR ('all cause mortality'/exp OR 'all cause mortality':ab,ti)))

Cohrance database:

#1	MeSH descriptor:[Medication Adherence] explode all trees	MeSH
#2	(medication adherence):ti,ab,kw	Limits
#3	(medication compliance):ti,ab,kw OR (medication persistence):ti,ab,kw OR (persistence):ti,ab,kw OR (compliance):ti,ab,kw	Limits
#4	#1 OR #2 OR #3	
#5	MeSH descriptor:[Antihypertensive Agent] explode all trees	MeSH
#6	MeSH descriptor:[Hydroxymethylglutaryl-CoA Reductase Inhibitors] explode all trees	MeSH
#7	MeSH descriptor:[Aspirin] explode all trees	MeSH
#8	MeSH descriptor:[Clopidogrel] explode all trees	MeSH
#9	MeSH descriptor:[Adrenergic beta-Antagonists] explode all trees	MeSH
#10	MeSH descriptor:[Hypoglycemic- Agent] explode all trees	MeSH

#11	#5 OR #6 OR #7 OR #8 OR #9 OR #10	Limits
#12	MeSH descriptor:[Cardiovascular Diseases] explode all trees	MeSH
#13	MeSH descriptor:[Coronary Artery Disease] explode all trees	MeSH
#14	MeSH descriptor:[Atherosclerosis] explode all trees	MeSH
#15	MeSH descriptor:[Coronary disease] explode all trees	MeSH
#16	MeSH descriptor:[Myocardial Infarction] explode all trees	MeSH
#17	MeSH descriptor:[Myocardial Ischemia] explode all trees	MeSH
#18	MeSH descriptor:[Stroke] explode all trees	MeSH
#19	MeSH descriptor:[Cerebrovascular Disease] explode all trees	MeSH
#20	MeSH descriptor:[Mortality] explode all trees	MeSH
#21	(all cause mortality):ti,ab,kw	MeSH
#22	#12 OR #13 OR #14 OR #15 OR #16 OR #17 OR #18 OR #19 OR #20 OR #21	Limits
#23	#4 AND #11 AND #22	Limits

S2. Reference list for the studies included

1. Korhonen MJ, Ruokoniemi P, Ilomäki J, Meretoja A, Helin-Salmivaara A, Huupponen R. Adherence to statin therapy and the incidence of ischemic stroke in patients with diabetes. Pharmacoepidemiol Drug Saf. 2016 Feb;25(2):161-9.
2. Bansilal S, Castellano JM, Garrido E, Wei HG, Freeman A, Spettell C, et al. Assessing the Impact of Medication Adherence on Long-Term Cardiovascular Outcomes. Journal of the American College of Cardiology. 2016;68(8):789-801.
3. Karlsson SA, Hero C, Svensson AM, Franzén S, Miftaraj M, Gudbjörnsdottir S, et al. Association between refill adherence to lipid-lowering medications and the risk of cardiovascular disease and mortality in Swedish patients with type 2 diabetes mellitus: A nationwide cohort study. BMJ Open. 2018;8(3).
4. Rodriguez F, Maron DJ, Knowles JW, Virani SS, Lin S, Heidenreich PA. Association of Statin Adherence With Mortality in Patients With Atherosclerotic Cardiovascular Disease. JAMA Cardiol. 2019 Mar 1;4(3):206-13.
5. Kim J, Lee HS, Nam CM, Heo JH. Effects of Statin Intensity and Adherence on the Long-Term Prognosis After Acute Ischemic Stroke. Stroke. 2017 Oct;48(10):2723-30.
6. Corrao G, Monzio Compagnoni M, Franchi M, Cantarutti A, Pugni P, Merlino L, et al. Good adherence to therapy with statins reduces the risk of adverse clinical outcomes even among very elderly. Evidence from an Italian real-life investigation. Eur J Intern Med. 2017 Jan;47:25-31.
7. Mathews R, Wang W, Kaltenbach LA, Thomas L, Shah RU, Ali M, et al. Hospital Variation in Adherence Rates to Secondary Prevention Medications and the Implications on Quality. Circulation. 2018 May 15;137(20):2128-38.
8. Yeo SH, Toh MPHS, Lee SH, Seet RCS, Wong LY, Yau WP. Impact of medication nonadherence on stroke recurrence and mortality in patients after first-ever ischemic stroke: Insights from registry data in Singapore. Pharmacoepidemiology and Drug Safety. 2020.
9. Rannanheimo PK, Tiittanen P, Hartikainen J, Helin-Salmivaara A, Huupponen R, Vahtera J, et al. Impact of Statin Adherence on Cardiovascular Morbidity and All-Cause Mortality in the Primary Prevention of Cardiovascular Disease: A Population-Based Cohort Study in Finland. Value in health : the journal of the International Society for Pharmacoeconomics and Outcomes Research. 2015 Sep;18(6):896-905.
10. Hickson RP, Robinson JG, Annis IE, Killeya-Jones LA, Fang G. It’s not too late to improve statin adherence: Association between changes in Statin adherence from before to after acute myocardial infarction and all-cause mortality. Journal of the American Heart Association. 2019;8(7).
11. Chen SH, Lee HC, Chang KC, Hung JW, Chen HM, Wu CY, et al. Low persistence of antithrombotic agents is associated with poor outcomes after first-ever acute ischemic stroke. Acta Neurologica Taiwanica. 2019;28(4):95-118.
12. Ye X, Qian C, Liu J, St. Peter WL. Lower risk of major cardiovascular events associated with adherence to colesevelam HCI. Pharmacotherapy. 2013;33(10):1062-70.
13. Molnar MZ, Gosmanova EO, Sumida K, Potukuchi PK, Lu JL, Jing J, et al. Predialysis Cardiovascular Disease Medication Adherence and Mortality After Transition to Dialysis. American Journal of Kidney Diseases. 2016;68(4):609-18.
14. Chen PS, Cheng CL, Yang YHK, Li YH. Statin adherence after ischemic stroke or transient ischemic

attack is associated with clinical outcome. Circulation Journal. 2016;80(3):731-7.
15. Phan DQ, Duan L, Lam B, Hekimian A, Wee D, Zadegan R, et al. Statin Adherence and Mortality in Patients Aged 80 Years and Older After Acute Myocardial Infarction. Journal of the American Geriatrics Society. 2019;67(10):2045-9.
16. Shalev V, Goldshtein I, Porath A, Weitzman D, Shemer J, Chodick G. Continuation of statin therapy and primary prevention of nonfatal cardiovascular events. The American journal of cardiology. 2012 Dec 15;110(12):1779-86.
17. Pittman DG, Chen W, Bowlin SJ, Foody JM. Adherence to statins, subsequent healthcare costs, and cardiovascular hospitalizations. The American journal of cardiology. 2011 Jun 1;107(11):1662-6.
18. Shalev V, Chodick G, Silber H, Kokia E, Jan J, Heymann AD. Continuation of statin treatment and all-cause mortality: a population-based cohort study. Archives of internal medicine. 2009 Feb 9;169(3):260-8.
19. Perreault S, Dragomir A, Blais L, Bérard A, Lalonde L, White M, et al. Impact of better adherence to statin agents in the primary prevention of coronary artery disease. European journal of clinical pharmacology. 2009 Oct;65(10):1013-24.
20. Corrao G, Scotti L, Zambon A, Baio G, Nicotra F, Conti V, et al. Cost-effectiveness of enhancing adherence to therapy with statins in the setting of primary cardiovascular prevention. Evidence from an empirical approach based on administrative databases. Atherosclerosis. 2011 Aug;217(2):479-85.
21. Rasmussen JN, Chong A, Alter DA. Relationship between adherence to evidence-based pharmacotherapy and long-term mortality after acute myocardial infarction. Jama. 2007 Jan 10;297(2):177-86.
22. Wei L, Flynn R, Murray GD, MacDonald TM. Use and adherence to beta-blockers for secondary prevention of myocardial infarction: who is not getting the treatment? Pharmacoepidemiology and drug safety. 2004 Nov;13(11):761-6.
23. Wei L, Wang J, Thompson P, Wong S, Struthers AD, MacDonald TM. Adherence to statin treatment and readmission of patients after myocardial infarction: a six year follow up study. Heart (British Cardiac Society). 2002 Sep;88(3):229-33.
24. Degli Esposti L, Saragoni S, Batacchi P, Benemei S, Geppetti P, Sturani A, et al. Adherence to statin treatment and health outcomes in an Italian cohort of newly treated patients: results from an administrative database analysis. Clinical therapeutics. 2012 Jan;34(1):190-9.
25. Perreault S, Ellia L, Dragomir A, Côté R, Blais L, Bérard A, et al. Effect of statin adherence on cerebrovascular disease in primary prevention. The American journal of medicine. 2009 Jul;122(7):647-55.
26. Mazzaglia G, Ambrosioni E, Alacqua M, Filippi A, Sessa E, Immordino V, et al. Adherence to antihypertensive medications and cardiovascular morbidity among newly diagnosed hypertensive patients. Circulation. 2009 Oct 20;120(16):1598-605.
27. Herttua K, Tabak AG, Martikainen P, Vahtera J, Kivimaki M. Adherence to antihypertensive therapy prior to the first presentation of stroke in hypertensive adults: population-based study. European heart journal. 2013 Oct;34(38):2933-9.
28. Corrao G, Parodi A, Nicotra F, Zambon A, Merlino L, Cesana G, et al. Better compliance to antihypertensive medications reduces cardiovascular risk. Journal of hypertension. 2011 Mar;29(3):610-8.
29. Krousel-Wood M, Holt E, Joyce C, Ruiz R, Dornelles A, Webber LS, et al. Differences in cardiovascular disease risk when antihypertensive medication adherence is assessed by pharmacy fill versus self-report: the Cohort Study of Medication Adherence among Older Adults (CoSMO). Journal of hypertension. 2015 Feb;33(2):412-20.
30. Wong MC, Tam WW, Cheung CS, Wang HH, Tong EL, Sek AC, et al. Drug adherence and the incidence of coronary heart disease- and stroke-specific mortality among 218,047 patients newly prescribed an antihypertensive medication: a five-year cohort study. International journal of cardiology. 2013 Sep 30;168(2):928-33.
31. Kim S, Shin DW, Yun JM, Hwang Y, Park SK, Ko YJ, et al. Medication Adherence and the Risk of Cardiovascular Mortality and Hospitalization Among Patients With Newly Prescribed Antihypertensive Medications. Hypertension (Dallas, Tex : 1979). 2016 Mar;67(3):506-12.
32. Herttua K, Martikainen P, Batty GD, Kivimaki M. Poor Adherence to Statin and Antihypertensive Therapies as Risk Factors for Fatal Stroke. Journal of the American College of Cardiology. 2016 Apr 5;67(13):1507-15.
33. Degli Esposti L, Saragoni S, Benemei S, Batacchi P, Geppetti P, Di Bari M, et al. Adherence to

antihypertensive medications and health outcomes among newly treated hypertensive patients. ClinicoEconomics and outcomes research : CEOR. 2011;3:47-54.
34. Yang Z, Howard DH, Will J, Loustalot F, Ritchey M, Roy K. Association of Antihypertensive Medication Adherence With Healthcare Use and Medicaid Expenditures for Acute Cardiovascular Events. Medical care. 2016 May;54(5):504-11.
35. Liu PH, Hu FC, Wang JD. Differential risks of stroke in pharmacotherapy on uncomplicated hypertensive patients? Journal of hypertension. 2009;27(1):174-80.
36. Corrao G, Rea F, Ghirardi A, Soranna D, Merlino L, Mancia G. Adherence with antihypertensive drug therapy and the risk of heart failure in clinical practice. Hypertension. 2015;66(4):742-9.
37. Yang Q, Chang A, Ritchey MD, Loustalot F. Antihypertensive Medication Adherence and Risk of Cardiovascular Disease Among Older Adults: A Population-Based Cohort Study. Journal of the American Heart Association. 2017 Jun 24;6(6).
38. Kim HJ, Yoon SJ, Oh IH, Lim JH, Kim YA. Medication adherence and the occurrence of complications in patients with newly diagnosed hypertension. Korean Circulation Journal. 2016;46(3):384-93.
39. Corrao G, Rea F, Monzio Compagnoni M, Merlino L, Mancia G. Protective effects of antihypertensive treatment in patients aged 85 years or older. Journal of hypertension. 2017 Jul;35(7):1432-41.
40. van Dongen MME, Aarnio K, Martinez-Majander N, Pirinen J, Sinisalo J, Lehto M, et al. Use of antihypertensive medication after ischemic stroke in young adults and its association with long-term outcome. Ann Med. 2019 Feb;51(1):68-77.
41. Lee HJ, Jang SI, Park EC. Effect of adherence to antihypertensive medication on stroke incidence in patients with hypertension: a population-based retrospective cohort study. BMJ open. 2017 Jul 2;7(6):e014486.
42. Kim J, Bushnell CD, Lee HS, Han SW. Effect of adherence to antihypertensive medication on the long-term outcome after hemorrhagic stroke in Korea. Hypertension (Dallas, Tex : 1979). 2018;72(2):391-8.
43. Lavie G, Hoshen M, Leibowitz M, Benis A, Akriv A, Balicer R, et al. Statin Therapy for Primary Prevention in the Elderly and Its Association with New-Onset Diabetes, Cardiovascular Events, and All-Cause Mortality. American Journal of Medicine. 2021;134(5):643-52.
44. Lee YR, Oh SS, Jang SI, Park EC. Statin adherence and risk of all-cause, cancer, and cardiovascular mortality among dyslipidemia patients: A time-dependent analysis. Nutrition, metabolism, and cardiovascular diseases : NMCD. 2020 Nov 27;30(12):2207-14.
45. Greenland M, Knuiman MW, Hung J, Nedkoff L, Arnet I, Rankin JM, et al. Cardioprotective medication adherence in Western Australians in the first year after myocardial infarction: restricted cubic spline analysis of adherence-outcome relationships. Scientific reports. 2020;10(1):4315.
46. Ahrens I, Khachatryan A, Monga B, Dornstauder E, Sidelnikov E. Association of Treatment Intensity and Adherence to Lipid-Lowering Therapy with Major Adverse Cardiovascular Events Among Post-MI Patients in Germany. BMJ open. 2021 May;38(5):2532-41.
